# Multifunctional Magnetic Ionic Liquid‐Carbon Nanohorn Complexes for Targeted Cancer Theranostics

**DOI:** 10.1002/smsc.202400640

**Published:** 2025-03-03

**Authors:** Yun Qi, Eijiro Miyako

**Affiliations:** ^1^ Graduate School of Advanced Science and Technology Japan Advanced Institute of Science and Technology 1‐1 Asahidai, Nomi Ishikawa 923‐1292 Japan

**Keywords:** cancer, carbon nanohorn, ionic liquid, nanoparticle, theranostics

## Abstract

Establishing a rational and simplified design for nanoparticles that selectively target and eliminate cancer cells is a crucial aspect of cancer treatment. In this study, a new multifunctional nanocomplex is developed based on a photoexothermic carbon nanohorn comprising the magnetic and anticancer ionic liquids 1‐butyl‐3‐methylimidazolium tetrachloroferrate and fluorescent indocyanine green, synthesized through a convenient sonication process. The synthesized nanocomplexes exhibit unique therapeutic, photothermal, magnetic, and fluorescent properties, enabling chemotherapeutic, light‐, and magnetic‐field‐driven cancer theranostics. Furthermore, this nanocomplex demonstrates prolonged water‐dispersing stability (7 days), high photothermal conversion efficiency (63%), and remarkable stability under biologically permeable near‐infrared laser irradiation. Simple magnetic guidance significantly enhances the accumulation of nanocomplexes at tumor sites, facilitating targeted delivery. In vitro and in vivo studies have demonstrated potent anticancer efficacy, high selective cytotoxicity against cancer cells, and minimal impact on normal tissues. This study represents the first application of magnetic ionic liquids in cancer treatment and provides a valuable platform for advanced nanotheranostics.

## Graphical abstract

A nanocomplex is prepared from the self‐organization of photoexothermic carbon nanohorn, magnetic and anticancer ionic liquid [Bmim][FeCl_4_], fluorescent indocyanine green, and biocompatible polyethylene glycol phospholipid (DSPE–PEG_2000_–NH_2_) by simple sonication. The chemo/photothermal/magnetic combined anticancer potential of the nanocomplex was evaluated in cancer cells and tumor‐bearing mice.

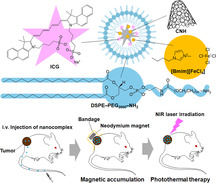

## 1 Introduction

Cancer is a highly invasive and metastatic disease characterized by abnormal cell proliferation. Despite significant scientific and technological advancements, it remains one of the most serious threats to human health globally.^[^ ^1^ ^]^ Primary treatment strategies for cancer include surgery, radiotherapy, and chemotherapy. However, because of the rapid growth of certain tumors and their indistinct boundaries with the surrounding healthy tissues, surgical intervention often requires the removal of a margin of normal tissue to ensure complete resection. Moreover, although radiotherapy and chemotherapy are effective, they lack specificity and may inadvertently damage healthy tissues, resulting in severe adverse side effects.^[^ ^2^, ^3^ ^]^

Photothermal therapy (PTT) has demonstrated substantial promise for cancer treatment, primarily because of its unique advantages of high specificity and minimal invasiveness. It uses near‐infrared (NIR) laser irradiation in combination with photothermal agents to generate localized heat, which leads to the thermal ablation of cancer cells. PTT also provides distinct benefits such as lower toxicity, enhanced specificity, and reduced invasiveness.^[^ ^4^, ^5^, ^6^, ^7^, ^8^ ^]^ These attributes make PTT a highly effective alternative for cancer treatment.

Materials such as gold, gallium, copper, semiconducting polymers, and carbon exhibit strong photothermal conversion capabilities in the NIR spectrum and are suitable photothermal agents for PTT to effectively eliminate tumors.^[^ ^9^, ^10^, ^11^, ^12^, ^13^ ^]^ In particular, carbon nanohorns (CNHs) have been used in PTT for tumor elimination because of their strong photothermal conversion capabilities, which demonstrate exceptional efficacy in the NIR region.^[^ ^14^, ^15^ ^]^ CNHs are composed of a single layer of graphene, similar to single‐walled carbon nanotubes. They have a distinct spherical structure with a diameter of ≈100 nm and are adorned with numerous short conical protrusions. This unique architecture facilitates the encapsulation of various molecules, endowing CNHs with excellent drug loading and targeted release capabilities.^[^ ^13^ ^]^ Furthermore, because of its biocompatibility and low cytotoxicity, CNHs are widely used in the biomedical field as a carrier for the delivery of drugs and antibodies, as well as in vivo bioimaging applications.^[^ ^16^, ^17^, ^18^, ^19^ ^]^ However, accurate delivery of CNHs in substantial quantities to tumors presents a significant challenge.

Because of the enhanced permeation and retention (EPR) effect, nanoparticles with diameters of 20–300 nm can accumulate in tumor tissues for long periods.^[^ ^20^ ^]^ Although the EPR effect can lead to an increased accumulation of nanoparticles in tumor tissues compared to normal tissues, it is difficult to achieve precise localization and enrichment of nanoparticles within solid tumors.^[^ ^21^ ^]^ This limits the broader application of nanoparticles, including CNHs, as novel functionalized nanomaterials for cancer treatment. To date, common strategies to overcome this obstacle include modifying the surface of nanoparticles to enhance their tumor‐targeting capabilities. For example, nanoparticles can be coated with biocompatible polymers such as polyvinyl alcohol or polyethylene glycol (PEG) to enhance their stability and dispersibility in vivo, further improving their efficacy and delivery properties.^[^ ^22^, ^23^, ^24^ ^]^ In addition, attaching ligands such as antibodies, peptides, or low‐molecular‐weight small molecules to the surface of nanoparticles can improve their affinity for tumor cells and enable targeted delivery within tumors.^[^ ^25^, ^26^, ^27^ ^]^ Nevertheless, the selectivity of nanoparticles against cancerous tumors is not yet sufficient. In addition, a simpler preparation process and rigid therapeutic strategies with high selectivity against tumors are essential for future clinical trials.

Ionic liquids are salts that exist in the liquid state under ambient conditions. They have various potential uses because of their remarkable features such as low flammability, negligible vapor pressure, ionic conductivity, chemical and thermal stability, and a wide liquid temperature range.^[^ ^28^, ^29^, ^30^, ^31^, ^32^, ^33^, ^34^, ^35^ ^]^ Recently, ionic liquids have been evaluated as anticancer agents and represent unique molecules for the treatment of various cancers;^[^ ^36^, ^37^, ^38^ ^]^ however, they represent a new type of therapy that requires further study. The magnetism of 1‐butyl‐3‐methylimidazolium tetrachloroferrate ([Bmim][FeCl_4_]) was first discovered by Hayashi et al.^[^ ^39^ ^]^ which opened new prospects for ionic liquids.^[^ ^40^ ^]^ Although most applications of magnetic ionic liquids have focused on extraction and separation processes,^[^ ^40^ ^]^ their application in cancer therapy is promising.

To explore a rational and simple design for precise cancer selectivity and control of nanomaterials, we developed a new class of functional nanocomplexes based on photoexothermic CNHs using magnetic and anticancer ionic liquids [Bmim][FeCl_4_], fluorescent indocyanine green (ICG), and biocompatible PEG phospholipids. This study not only represents the first attempt to apply a magnetic ionic liquid to the field of cancer treatment but also establishes a new cancer theranostic platform through the rational molecular design of nanocomplexes. By simply applying a neodymium magnet to the tumor site, the nanocomplexes could be effectively guided to the tumor site with bright NIR fluorescence (FL) under the influence of an external magnetic field. The higher accumulation of these nanocomplexes at tumor sites may enhance the powerful photothermal conversion ability of CNHs while exploiting the potent anticancer properties of [Bmim][FeCl_4_], thereby achieving effective anticancer theranostics. By using the potential of smart nanocomplexes, we have developed a new strategy for precise and effective cancer treatment.

## 2 Results and Discussion

### 2.1 Characterization of Nanocomplexes

**Figure 1:**
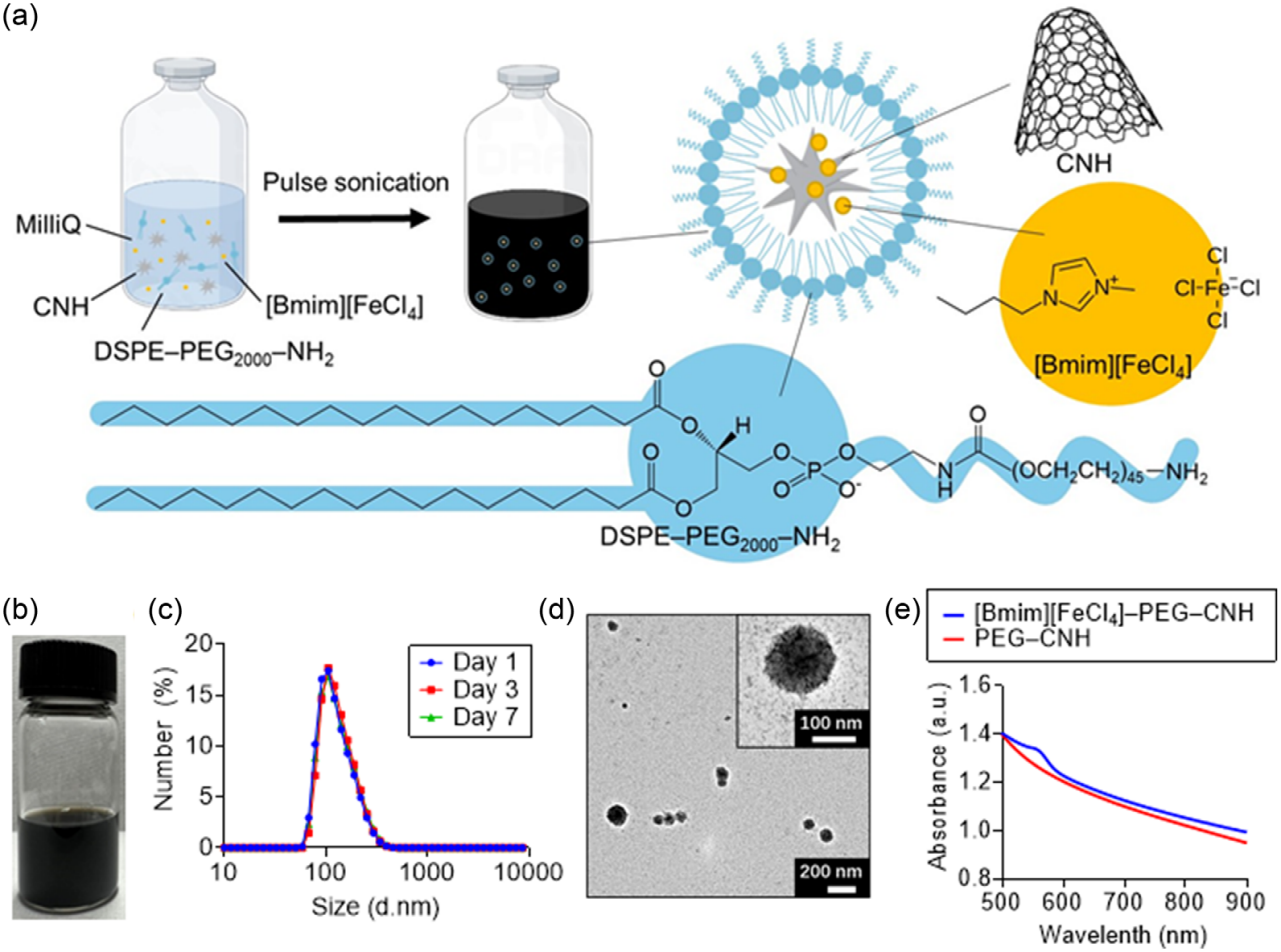
Structural characterization of functional CNH nanoparticles. a) Schematic illustration of the preparation of [Bmim][FeCl_4_]–PEG–CNH nanocomplexes. Sonication‐promoted nanoparticularization and surface modification of DSPE–PEG_2000_–NH_2_ and [Bmim][FeCl_4_] on CNH. b) Image of the prepared [Bmim][FeCl_4_]–PEG–CNH aqueous solution. c) Dynamic light scattering (DLS) size distribution of [Bmim][FeCl_4_]–PEG–CNH. d) Transmission electron microscopy (TEM) images of [Bmim][FeCl_4_]–PEG–CNH. High‐magnification image in the upper right panel. e) Ultraviolet–visible–near‐infrared (UV–vis–NIR) absorbance spectra of an aqueous solution of [Bmim][FeCl_4_]–PEG–CNH (CNH concentration = 25 μg mL^−1^, [Bmim][FeCl_4_] concentration = 5 μL mL^−1^), and PEG–CNH (CNH concentration = 25 μg mL^−1^).

CNHs are inherently insoluble in water; however, their dispersibility in water can be enhanced by surface modification.^[^ ^14^, ^15^, ^16^, ^17^, ^18^, ^19^ ^]^ [Bmim][FeCl_4_] also exhibits strong hydrophobic properties.^[^ ^39^, ^40^ ^]^ We designed a simple and effective preparation method for a highly water‐dispersible magnetic [Bmim][FeCl_4_]‐modified CNH nanocomplex using 1,2‐distearoyl‐sn‐glycero‐3‐phosphoethanolamine‐N‐[amino(polyethylene glycol)‐2000] (DSPE–PEG_2000_–NH_2_), which was selected owing to its high biocompatibility and versatibility.^[^ ^14^ ^]^ Using conventional pulsed sonication, we synthesized a nanocomplex with excellent water dispersibility (**Figure** **1**a). Although various decorations of nanocarbon surfaces with solid‐state magnetic nanoparticles have been reported, multistep procedures are required.^[^ ^41^, ^42^ ^]^ However, the water dispersibility of ordinary magnetic nanocarbons is very low because solid‐state magnetic nanoparticles tend to aggregate strongly with one another in dispersions. The prepared [Bmim][FeCl_4_]–PEG–CNH suspension was dark in color (Figure 1b) and remained stable in water at room temperature for at least 1 week, based on dynamic light scattering (DLS) measurements (Figure 1c). Transmission electron microscopy (TEM) images revealed the morphology and particle size (diameter ≈ 120 nm) of [Bmim][FeCl_4_]–PEG–CNH, which is consistent with the DLS results (Figure 1d). Although pristine CNHs did not have any nanolayers, PEG–CNH contained particles of homogeneous spherical morphology and surface nanolayers because of the PEG coating (Figure S1, Supporting Information).

To characterize the optical properties of the resulting nanocomplexes, the ultraviolet–visible–NIR (UV–vis–NIR) absorption spectra of the [Bmim][FeCl_4_]–PEG–CNH and PEG–CNH suspensions were captured (Figure 1e), which showed that the absorption of both samples occurred in the NIR region. We also observed a linear increase in the NIR optical absorbance at 808 nm with increasing concentrations of [Bmim][FeCl_4_]–PEG–CNH in the aqueous solution (Figure S2, Supporting Information). A characteristic peak of the FeCl_4_ anion appeared in the spectrum of [Bmim][FeCl_4_]–PEG–CNH at ≈560 nm because of the conjugation of [Bmim][FeCl_4_] with CNH.^[^ ^43^ ^]^ We estimated that most [Bmim][FeCl_4_] molecules could be loaded onto the surface of CNH through hydrophobic and –π interactions,^[^ ^44^ ^]^ because no phase separation of the ionic liquid was observed in the prepared vials and no aggregation was observed in the DLS results. Overall, these data suggest that PEG‐modified CNH nanoparticles can effectively encapsulate [Bmim][FeCl_4_] while maintaining their dispersion stability. This highlights their potential as light‐responsive photothermal agents for cancer theranostics. We also believe that anionic [FeCl_4_] molecules were loaded onto CNH via ionic interactions with cationic [Bmim] molecules. Thermogravimetric analysis (TGA) showed that ≈0.98 mg of PEG and 273 mg of [Bmim][FeCl_4_] were coated onto the surface of 1 mg of CNHs (Figure S3, Supporting Information). The loading efficiencies of PEG and [Bmim][FeCl_4_] on the CNHs were 98 and 99%, respectively. Using a UV–vis–NIR spectrometer, we confirmed that 98% of [Bmim][FeCl_4_] remained within the nanocomplexes, even after filtration (Figure S4, Supporting Information). These results clearly indicate that the [Bmim][FeCl_4_] and PEG molecules were attached to the surface of the CNH.

**Figure 2:**
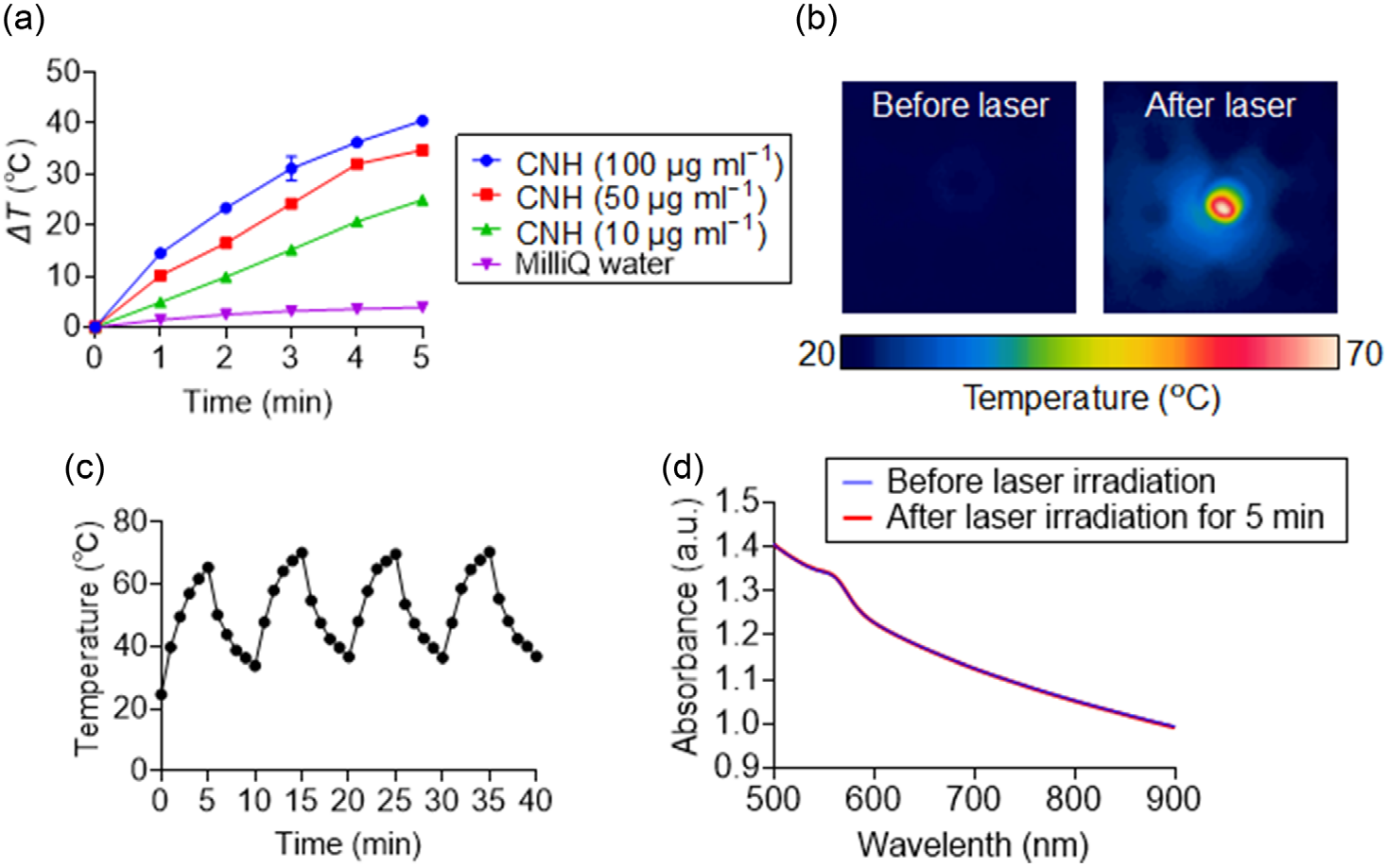
Photothermal conversion evaluation of laser‐induced CNH nanocomplexes. a) Laser‐induced temperature increase in Milli‐Q water (control) and [Bmim][FeCl_4_]–PEG–CNH suspensions at various CNH concentrations. Data are presented as mean ± standard error of the mean (SEM) (*n* = 3; independen*t* tests). b) IR thermal images of the [Bmim][FeCl_4_]–PEG–CNH suspension (CNH concentration = 100 μg mL^−1^) exposed to 808 nm laser irradiation at 0.7 W (≈35.6 mW mm^−2^) for 5 min. c) Stability of the [Bmim][FeCl_4_]–PEG–CNH suspension under photothermal heating and natural cooling cycles exposed to 808 nm laser irradiation at 0.7 W (≈35.6 mW mm^−2^) power. (CNH concentration = 100 μg mL^−1^ in 1 mL MilliQ water). d) Ultraviolet–visible–near‐infrared (UV–vis–NIR) absorbance spectra of [Bmim][FeCl_4_]–PEG–CNH before and after laser irradiation at 0.7 W (≈35.6 mW mm^−2^) for 5 min.

Subsequently, the photothermal conversion capability of [Bmim][FeCl_4_]–PEG–CNH was evaluated based on its absorbance in the NIR region. Under 808 nm NIR laser irradiation, the temperature increase (*ΔT*) at a power of 0.7 W (≈35.6 mW mm^−2^) was measured for various concentrations of [Bmim][FeCl_4_]–PEG–CNH suspensions using a thermocouple. The *ΔT* of each [Bmim][FeCl_4_]–PEG–CNH suspension increased significantly with prolonged laser irradiation, whereas the Milli‐Q water control exhibited almost no increase in temperature (**Figure** **2**a). By adjusting the laser power and concentration of the [Bmim][FeCl_4_]–PEG–CNH suspension, the temperature was readily controlled, thereby enhancing the operability and precision of [Bmim][FeCl_4_]–PEG–CNH for PTT (Figure S5, Supporting Information). Infrared (IR) thermographic images revealed obvious temperature changes in the [Bmim][FeCl_4_]–PEG–CNH suspension after laser irradiation (Figure 2b). These images support similar conclusions, showing that at a laser power of 0.7 W (≈35.6 mW mm^−2^), the temperature of a 100 μg mL^−^1 [Bmim][FeCl_4_]–PEG–CNH suspension was significantly increased, reaching ≈69 °C after 5 min of irradiation. Furthermore, suspensions with different concentrations of [Bmim][FeCl_4_]–PEG–CNH (10 and 50 μg mL^−1^) exposed to a lower laser power of 0.3 W (≈15.3 mW mm^−2^) also exhibited varying temperature increases after 5 min of irradiation, demonstrating concentration‐dependent photothermal effects (Figure S6, Supporting Information).

The photothermal stability of [Bmim][FeCl_4_]–PEG–CNH was further evaluated using a laser on/off cycling test (Figure 2c). Under 0.7 W (≈35.6 mW mm^−2^) NIR laser irradiation, [Bmim][FeCl_4_]–PEG–CNH underwent four cycles of heating and natural cooling. The maximum temperature attained after each cycle of laser irradiation remained consistent, demonstrating excellent photothermal stability of the nanocomplexes. Notably, from the second cycle onwards, the maximum temperatures were slightly higher than those in the first cycle. This may be attributed to incomplete cooling after the first cycle, in which the temperature at the end of the cooling phase (10 min) did not return to the initial baseline (0 min). Nonetheless, this discrepancy disappeared in the subsequent cycles. More importantly, the UV–Vis–NIR optical absorption spectra of the [Bmim][FeCl_4_]–PEG–CNH suspension before and after laser irradiation showed no change in absorbance, confirming that the nanocomplex did not undergo degradation during NIR laser exposure (Figure 2d).

The photothermal conversion efficiency of [Bmim][FeCl_4_]–PEG–CNH at 808 nm was ≈63%. In comparison, other photothermal nanomaterials, such as metal‐based materials, carbon dots, and semiconductor polymer nanoparticles, exhibited lower conversion efficiencies than [Bmim][FeCl_4_]–PEG–CNH (Table S1, Supporting Information).^[^ ^12^, ^45^, ^46^, ^47^ ^]^ This superior efficiency highlights the potential of [Bmim][FeCl_4_]–PEG–CNH as a highly effective photothermal agent for cancer treatment.

**Figure 3:**
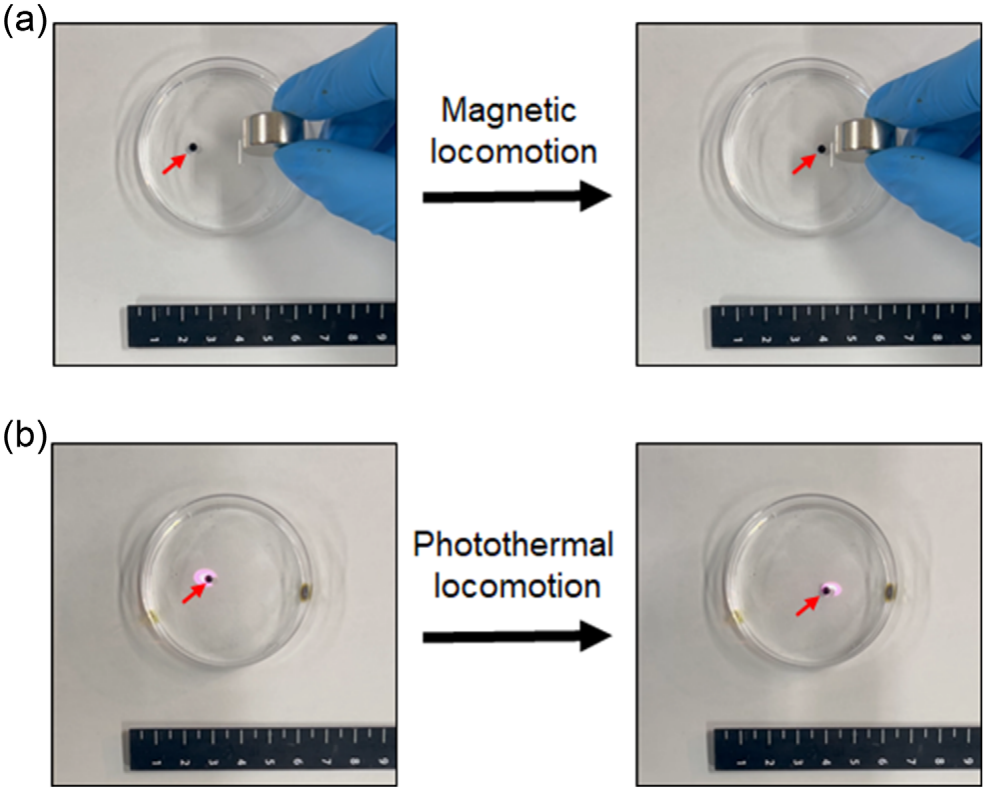
Controllable movement of a [Bmim][FeCl_4_]–PEG–CNH droplet by a) magnet and b) 808 nm laser irradiation at 0.7 W (≈35.6 mW mm^−2^). The red arrows indicate the location of the [Bmim][FeCl_4_]–PEG–CNH droplet.

The magnetic ionic liquid [Bmim][FeCl_4_] exhibits a strong response to external magnetic fields.^[^ ^41^, ^42^ ^]^ By exploiting this property, we examined the ability of [Bmim][FeCl_4_]–PEG–CNH droplets in a fluorous solvent to move under a magnetic field (**Figure** **3**a and Video S1, Supporting Information). When a neodymium magnet was placed above the [Bmim][FeCl_4_]–PEG–CNH droplet, the droplet moved toward the magnet. The trajectory of the droplet could be precisely controlled by adjusting the position of the magnet. Moreover, when irradiated with a 0.7 W NIR laser, the powerful photothermal conversion ability of CNHs resulted in thermal convection at the air–liquid interface, which caused dynamic motion of the [Bmim][FeCl_4_]–PEG–CNH droplet in a fluorous solvent (Figure 3b and Video S2, Supporting Information). The temperature of the [Bmim][FeCl_4_]–PEG–CNH droplet reached 36.5 °C from ≈21.0 °C just after laser irradiation (Figure S7, Supporting Information). In contrast, the [Bmim][FeCl_4_] droplet could not be moved by laser irradiation because of the absence of photothermal conversion and absorbance in the NIR region (Video S3, Figure S7 and S8, Supporting Information). A surface tension gradient (thermal convection) was generated using a temperature gradient. The natural flow of liquids based on different surface tensions results in their spontaneous movement.^[^ ^48^, ^49^ ^]^ Nonetheless, this combination of magnetic and photothermal control offers the potential for the precise manipulation of [Bmim][FeCl_4_]–PEG–CNH within a living biological body, which opens new possibilities for applications in targeted therapy and controlled delivery.

### 2.2 In vitro anticancer efficacy

**Figure 4:**
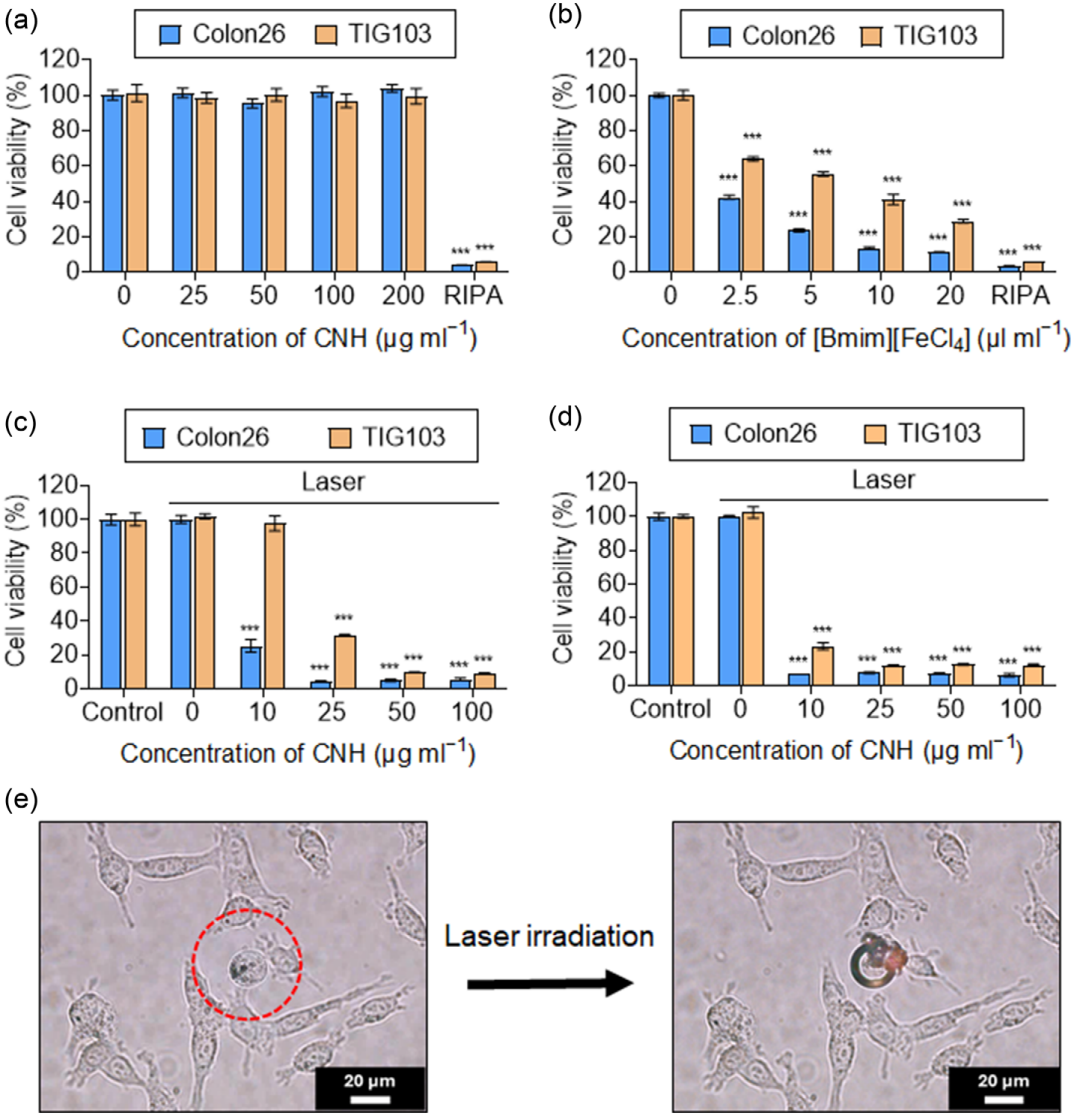
Laser‐induced cytotoxicity of CNH nanocomplexes. Viability of TIG103 and Colon26 cells treated with RIPA buffer (control) and a) PEG–CNH at various CNH concentrations and b) [Bmim][FeCl_4_]–PEG–CNH at various [Bmim][FeCl_4_] concentrations. Data are presented as the mean ± standard error of the mean (SEM) (*n* = 5; biologically independent tests), ***p < 0.001 versus the control without nanoparticles (Student's *t*‐test). c) Laser‐induced [Bmim][FeCl_4_]–PEG–CNH cytotoxicity evaluation in TIG103 and Colon26 cells with 5 min laser irradiation [0.7 W (≈35.6 mW mm^−2^] at various CNH concentrations. Data are presented as the mean ± SEM (*n* = 5; biologically independen*t* tests), ***p < 0.001 versus the control without laser irradiation (Student's *t*‐test). Concentrations of [Bmim][FeCl_4_] are 0, 2, 5, 10, and 20 μL mL^−1^ against each concentration of CNH (0, 10, 25, 50, and 100 μg mL^−1^), respectively. d) Laser‐induced [Bmim][FeCl_4_]–PEG–CNH cytotoxicity evaluation in TIG103 and Colon26 cells after 5 min of laser irradiation under a magnetic field at the bottom of the plate well containing various CNH concentrations. Concentrations of [Bmim][FeCl_4_] are 0, 2, 5, 10, and 20 μL mL^−1^ against each concentration of CNH (0, 10, 25, 50, 100 μg mL^−1^), respectively. Data are presented as the mean ± SEM (*n* = 5; biologically independen*t* tests), ***p < 0.001 versus the control without laser irradiation (Student's *t*‐test). e) Colon26 cancer cell destruction by laser‐induced [Bmim][FeCl_4_]–PEG–CNH before and after laser irradiation [wavelength = 808 nm, laser power = 254 mW (≈129 mW mm^−2^)]. The red dashed circle represents the location of the laser irradiation.

The anticancer efficacy of the synthesized nanocomplexes was evaluated using normal diploid fibroblasts (TIG103) and murine colon carcinoma (Colon26) cells. The cells were incubated for 24 h with various concentrations of PEG–CNH and [Bmim][FeCl_4_]–PEG–CNH. PEG–CNH suspensions at different concentrations exhibited no cytotoxicity toward TIG103 or Colon26 cells (**Figure** **4**a). The high biocompatibility and low toxicity of CNH has been confirmed by several research groups.^[^ ^17^, ^18^, ^19^, ^20^ ^]^ In contrast, after encapsulating the ionic liquid, [Bmim][FeCl_4_]–PEG–CNH exhibited cytotoxicity in both cell lines. [Bmim][FeCl_4_]–PEG–CNH exhibited stronger cytotoxic effects against Colon26 cancer cells than TIG103 cells, likely because of the specificity of conventional nonmagnetic ionic liquids toward cancer cells (Figure 4b).^[^ ^50^, ^51^, ^52^ ^]^ In general, normal cells have healthy cellular and nuclear membranes and rigid protein and mitochondrial structures compared to cancer cells, which are continuously and destructively proliferating. Therefore, ionic liquids may directly interact with and destroy such fragile and deregulated cancer cells and cancer nuclear membranes through several different mechanisms, such as alteration of cell membrane viscoelasticity and lipid distribution, mitochondrial dysfunction and permeabilization, disruption of cellular and nuclear membranes, production of reactive oxygen species, changes in the functions of transmembrane and cytoplasmic proteins and enzymes, and fragmentation of DNA.^[^ ^50^, ^51^, ^52^ ^]^ For both nanocomplexes, radioimmunoprecipitation assay (RIPA) lysis buffer was used as a positive control, which consistently induced strong cytotoxicity in both cell lines.

The cytotoxicity of the laser‐induced [Bmim][FeCl_4_]–PEG–CNH nanocomplexes was further examined. TIG103 and Colon26 cells co‐incubated with either PEG–CNH or [Bmim][FeCl_4_]–PEG–CNH were partially eliminated following exposure to 808 nm NIR laser irradiation at 0.7 W (≈35.6 mW mm^−2^) for 5 min because of the strong photothermal conversion properties of CNHs (Figure 4c). By placing neodymium magnets at the bottom of the plate under the influence of a magnetic field, [Bmim][FeCl_4_]–PEG–CNH accumulated more effectively in the cells, leading to enhanced cell elimination (Figure 4d). The laser‐induced cytotoxicity of [Bmim][FeCl_4_]–PEG–CNH was significantly lower against normal TIG103 cells than Colon26 cancer cells. This may be attributed to the inherent heat sensitivity of cancer cells, which arises from selective biochemical responses, such as the activation of heat‐shock proteins and unique molecular signaling pathways under thermal stress.^[^ ^53^ ^]^ These results further highlight the selective anticancer efficacy of laser‐induced [Bmim][FeCl_4_]–PEG–CNH and the increased accumulation of the nanocomplexes under an external magnetic field.

To validate the laser‐induced cytotoxicity of the [Bmim][FeCl_4_]–PEG‐CNH nanocomplexes, their real‐time anticancer activity was evaluated using a single‐laser beam integrated into an FL microscope. Following 808 nm laser irradiation at 254 mW (≈129 mW mm^−2^), the structure of the Colon26 cancer cells was immediately disrupted, forming bubbles, which probably resulted from water vaporization caused by the strong photothermal conversion of CNHs (Figure 4e and Video S4, Supporting Information). In contrast, the control group without [Bmim][FeCl_4_]–PEG–CNH exhibited no cancer cell destruction, confirming that the photothermal effect was specific to the nanocomplexes (Figure S9 and Video S5, Supporting Information). These results are consistent with previous findings, further demonstrating that laser‐activated [Bmim][FeCl_4_]–PEG–CNH nanocomplexes can precisely target and eliminate cancer cells, thereby elucidating their potential as effective agents for PTT.

### 2.3 In vivo cancer theranostic

**Figure 5:**
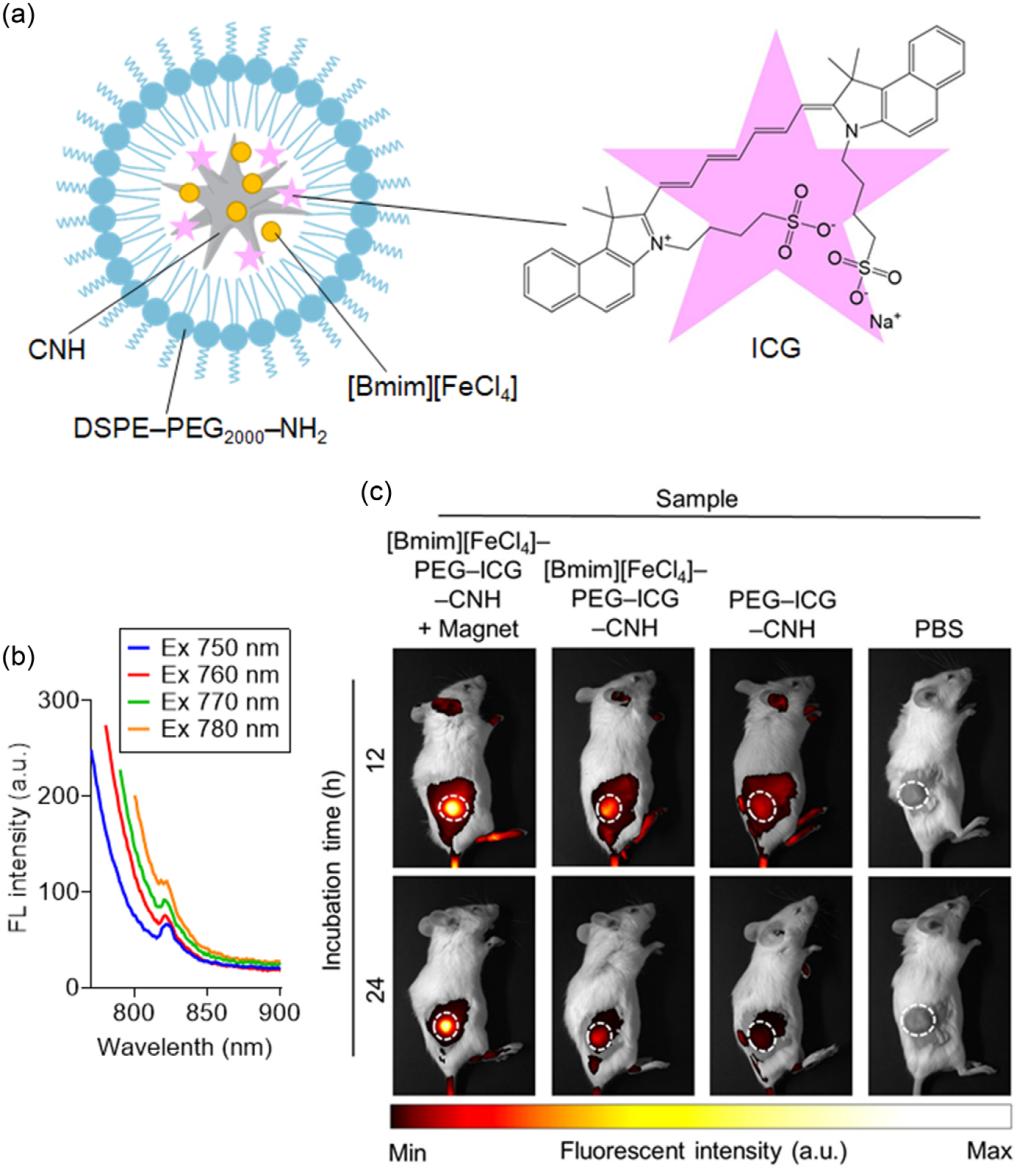
Biological distribution of the nanocomplexes. a) Schematic illustration of [Bmim][FeCl_4_]–PEG–ICG–CNH nanocomplexes. b) Fluorescence (FL) spectra of [Bmim][FeCl_4_]–PEG–ICG–CNH at different excitation wavelengths (ICG concentration = 125 μg mL^−1^, CNH concentration = 125 μg mL^−1^). c) FL imaging of Colon26 tumor‐bearing mice following an intravenous injection of [Bmim][FeCl_4_]–PEG–ICG–CNH, PEG–ICG–CNH, and PBS (ICG concentration = 1 mg mL^−1^, CNH concentration = 1 mg mL^−1^). White dashed circles denote solid tumor locations.

To assess the tumor‐targeting capability of the [Bmim][FeCl_4_]–PEG–CNH nanocomplexes, a fluorescent ICG probe was encapsulated to synthesize [Bmim][FeCl_4_]–PEG–ICG–CNH nanocomplexes (**Figure** **5**a). FL spectrometry revealed that the synthesized [Bmim][FeCl_4_]–PEG–ICG–CNH exhibited NIR FL emission at various NIR excitation wavelengths (Figure 5b), whereas [Bmim][FeCl_4_]–PEG–CNH did not exhibit such FL emission (Figure S10, Supporting Information). To determine the systemic pharmacokinetics of [Bmim][FeCl_4_]–PEG–ICG–CNH in vivo, the FL distribution was monitored in mice using NIR bioimaging. The distribution of the nanocomplexes was observed over time in tumors following intravenous (i.v.) injection through the tail vein of Colon26 tumor‐bearing mice (Figure 5c). Because of the EPR effect,^[^ ^20^ ^]^ passive accumulation of [Bmim][FeCl_4_]–PEG–ICG–CNH was gradually observed at the tumor site over time. In mice with neodymium magnets fixed at the tumor site, a higher accumulation of nanocomplexes was observed, accompanied by a stronger FL intensity. In contrast, mice injected with PEG–ICG–CNH and [Bmim][FeCl_4_]–PEG–ICG–CNH without neodymium magnets exhibited similar FL distributions, whereas the phosphate‐buffered saline (PBS) control group showed no FL.

Minor amounts of FL were detected in the liver and kidneys after dissection, suggesting that the primary elimination pathway for nanocomplexes occurs through renal and hepatic metabolism. FL observed in the lungs may be attributed to pulmonary extraction by immune cells, such as alveolar macrophages (Figure S11, Supporting Information).^[^ ^54^, ^55^ ^]^ Nanoparticle‐mediated pulmonary drug delivery has gained increasing attention as a strategy to overcome biological barriers and achieve site‐specific drug delivery by controlling the release of loaded drug(s) at target sites.^[^ ^56^ ^]^ Nonetheless, these findings conclusively demonstrate that [Bmim][FeCl_4_]–PEG–ICG–CNH nanocomplexes exhibit exceptional in vivo targeting abilities and can effectively accumulate at tumor sites under magnetic control.

The lack of in vivo toxicity of the [Bmim][FeCl_4_]–PEG–ICG–CNH nanocomplexes was further confirmed by blood tests (Table S2, Supporting Information). After seven days, there was no statistically significant difference in complete blood counts or biochemical parameters of mice intravenously injected with PBS or the [Bmim][FeCl_4_]–PEG–ICG–CNH suspension. Furthermore, [Bmim][FeCl_4_]–PEG–ICG–CNH did not exhibit any toxicity in tissues 7 days after i.v. injection (Figure S12, Supporting Information). Hematoxylin and eosin (H&E) staining analyses demonstrated that after i.v. injection of [Bmim][FeCl_4_]–PEG–ICG–CNH, the tissues resembled those of the control group (PBS buffer).

**Figure 6:**
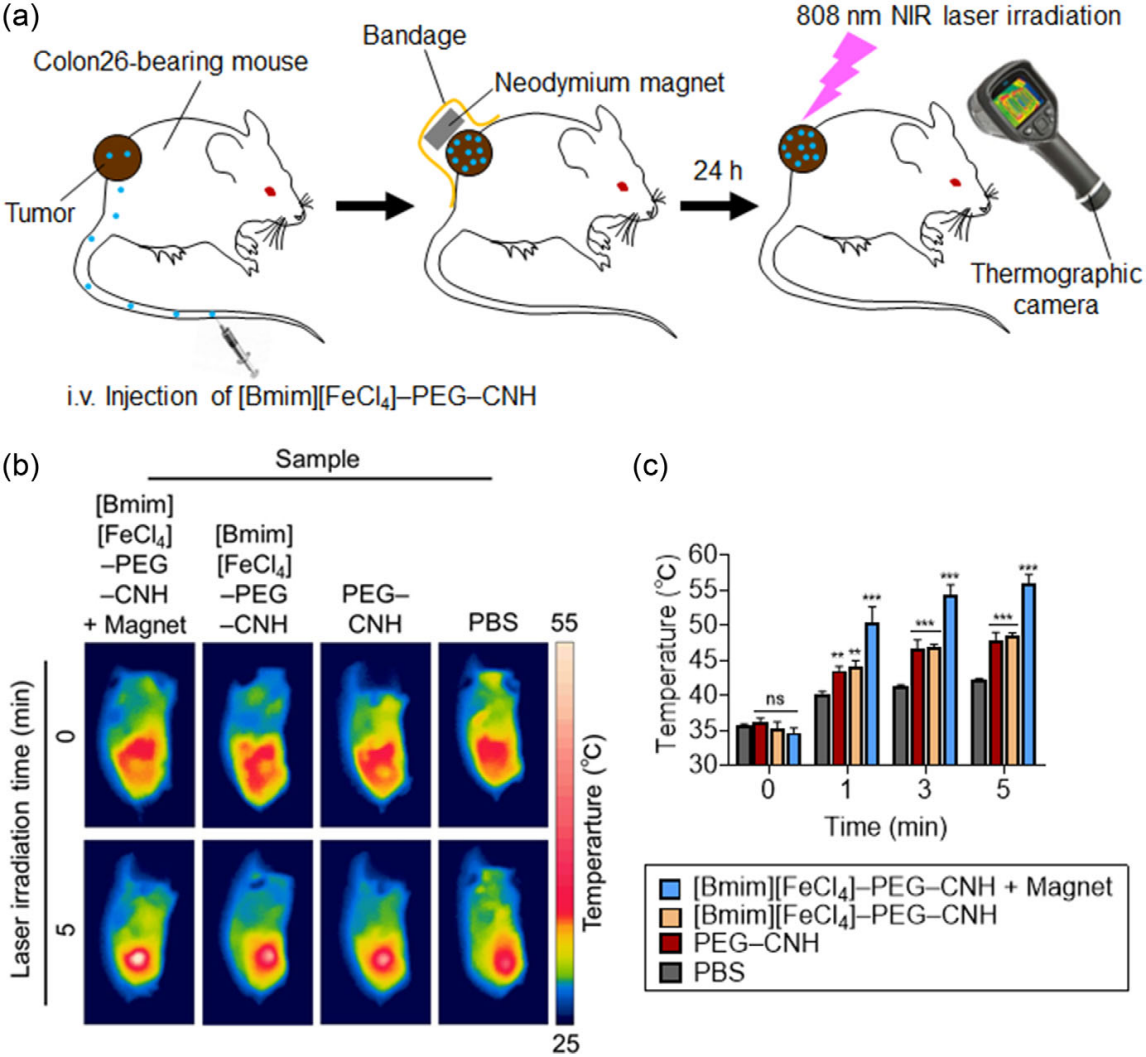
In vivo photothermal conversion activity of laser‐ and magnetic‐field‐induced Bmim FeCl_4_–PEG–CNH. a) Experimental design of the in vivo anticancer experiment. PBS or a suspension of [Bmim][FeCl_4_]–PEG–CNH or PEG–CNH was intravenously injected into Colon26‐bearing mice. A neodymium magnet is placed on the tumor using a bandage. After 24 h, the neodymium magnets are removed and the tumors were exposed to 808 nm laser irradiation [0.7 W (≈35.6 mW mm^−2^) for 5 min each day (a total of four times)]. b) Thermographic images of the tumor at the body surface after treatment with 808 nm laser‐irradiated Bmim FeCl_4_–PEG–CNH, PEG–CNH or PBS [Laser power = 0.7 W (≈35.6 mW mm^−2^), irradiation time = 5 min]. c) Solid tumor surface temperature of Colon26‐bearing mice after intravenous injection with Bmim FeCl_4_–PEG–CNH, PEG–CNH, or PBS followed by 808 nm laser irradiation for 5 min [laser power = 0.7 W (≈35.6 mW mm^−2^)]. Data are expressed as the mean ± standard error of the mean (SEM); *n* = 5 independent experiments. Statistical significance is calculated compared to the PBS group. ns, not significant; **p < 0.01; and ***p < 0.001, by Student's *t*‐test.

After confirming the in vivo targeting performance and controllability of the [Bmim][FeCl_4_]–PEG–CNH nanocomplexes, their laser‐induced photothermal conversion properties were evaluated using a homologous tumor model. Following the i.v. injection of the nanocomplexes, neodymium magnets were affixed to the solid tumors using a medical bandage. After 24 h, the solid tumors were exposed to an 808 nm NIR laser at 0.7 W (≈35.6 mW mm^−2^) for 5 min. During laser irradiation, the surface temperatures of the solid tumors were continuously monitored using a thermographic camera (**Figure** **6**a,b). The surface temperature of the solid tumors in all mice was ≈35 °C before laser irradiation. However, after 5 min of NIR laser irradiation, the surface temperature of the tumors of mice injected with [Bmim][FeCl_4_]–PEG–CNH and after magnetic attraction increased significantly, reaching ≈56 °C. In contrast, tumors of mice injected with PEG–CNH or [Bmim][FeCl_4_]–PEG–CNH without magnetic induction exhibited a lower increase in temperature, reaching ≈48 °C. In addition, even PBS‐injected mice showed a slight temperature increase under NIR laser irradiation, which was likely the result of the conversion of light energy to heat in the skin, blood, and tissues.

**Figure 7:**
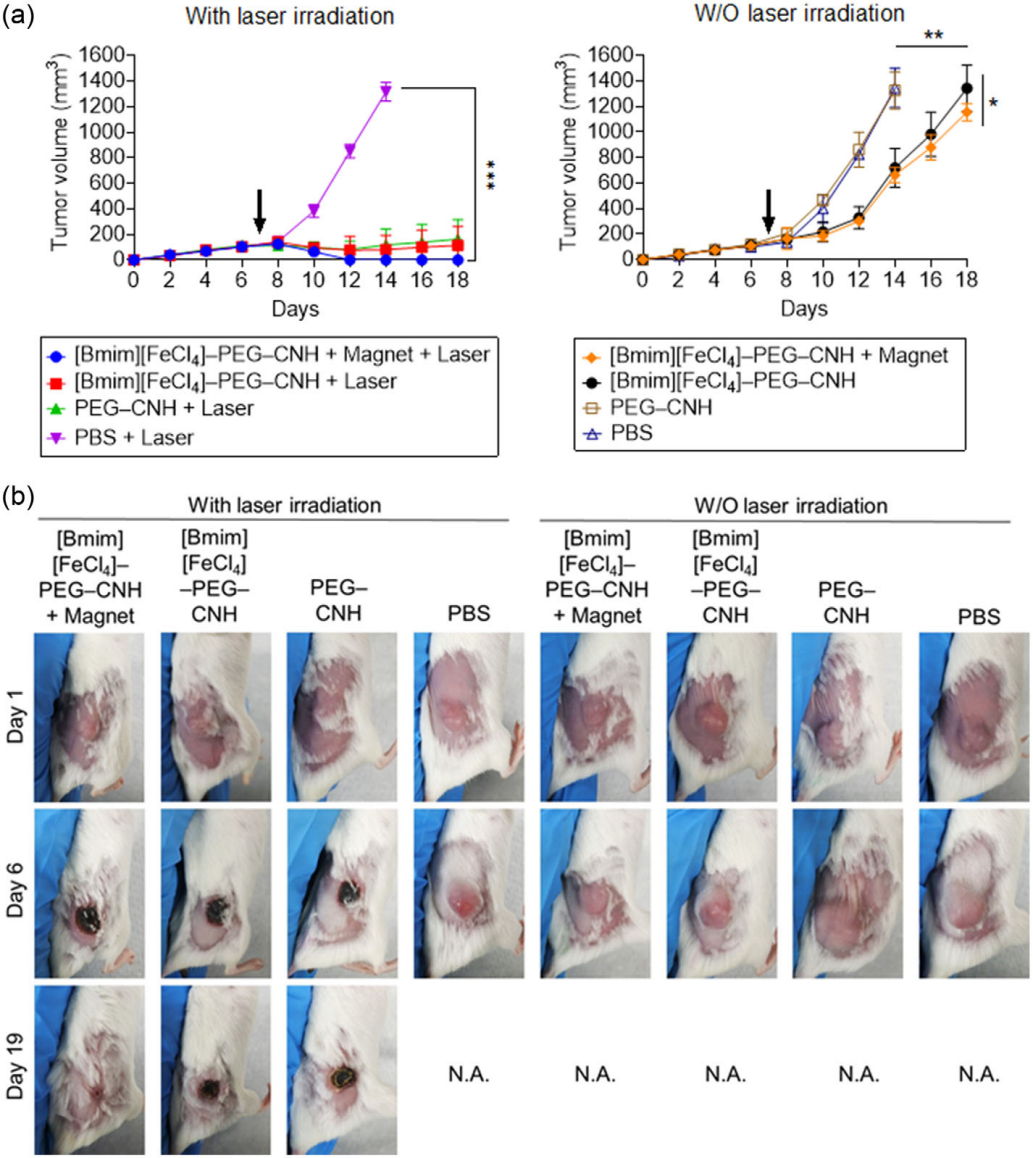
In vivo anticancer efficacy of light‐ and magnetic field‐activatable [Bmim][FeCl_4_]–PEG–CNH nanocomplexes. a) Anticancer effects of various samples after each treatment. Data are expressed as the mean ± standard error of the mean (SEM) (*n* = 5 biologically independen*t* tests). *p < 0.05, **p < 0.01, and ***p < 0.001, by Student's *t*‐test. Black arrows indicate the time point of sample administration. b) Images of mice after each treatment.

The synthesized CNH nanocomplexes exhibited significant anticancer effects under daily NIR laser irradiation (**Figure** **7**a). In mice injected with [Bmim][FeCl_4_]–PEG–CNH after magnetic induction, complete tumor elimination was observed after the sixth irradiation, achieving a 100% cure rate. No recurrence was observed during the following 20 days (Figure 7a), although slight burns were observed on the skin.

At the tumor site, these burn scars eventually fell off during subsequent tests (Figure 7b). In contrast, while the solid tumor volumes of mice injected with PEG–CNH or [Bmim][FeCl_4_]–PEG–CNH without magnetic attraction were generally controlled by NIR laser irradiation, with a few tumors being cured, tumor regrowth occurred once the laser treatment was stopped. This suggests the problem of relying solely on the EPR effect for tumor targeting. In the control group that did not receive NIR laser irradiation, the anticancer properties of the ionic liquids showed some effects. The growth rate of solid tumors in mice injected with [Bmim][FeCl_4_]–PEG–CNH decreased, with slower tumor growth observed in mice in which the magnets were fixed at the tumor site.

**Figure 8:**
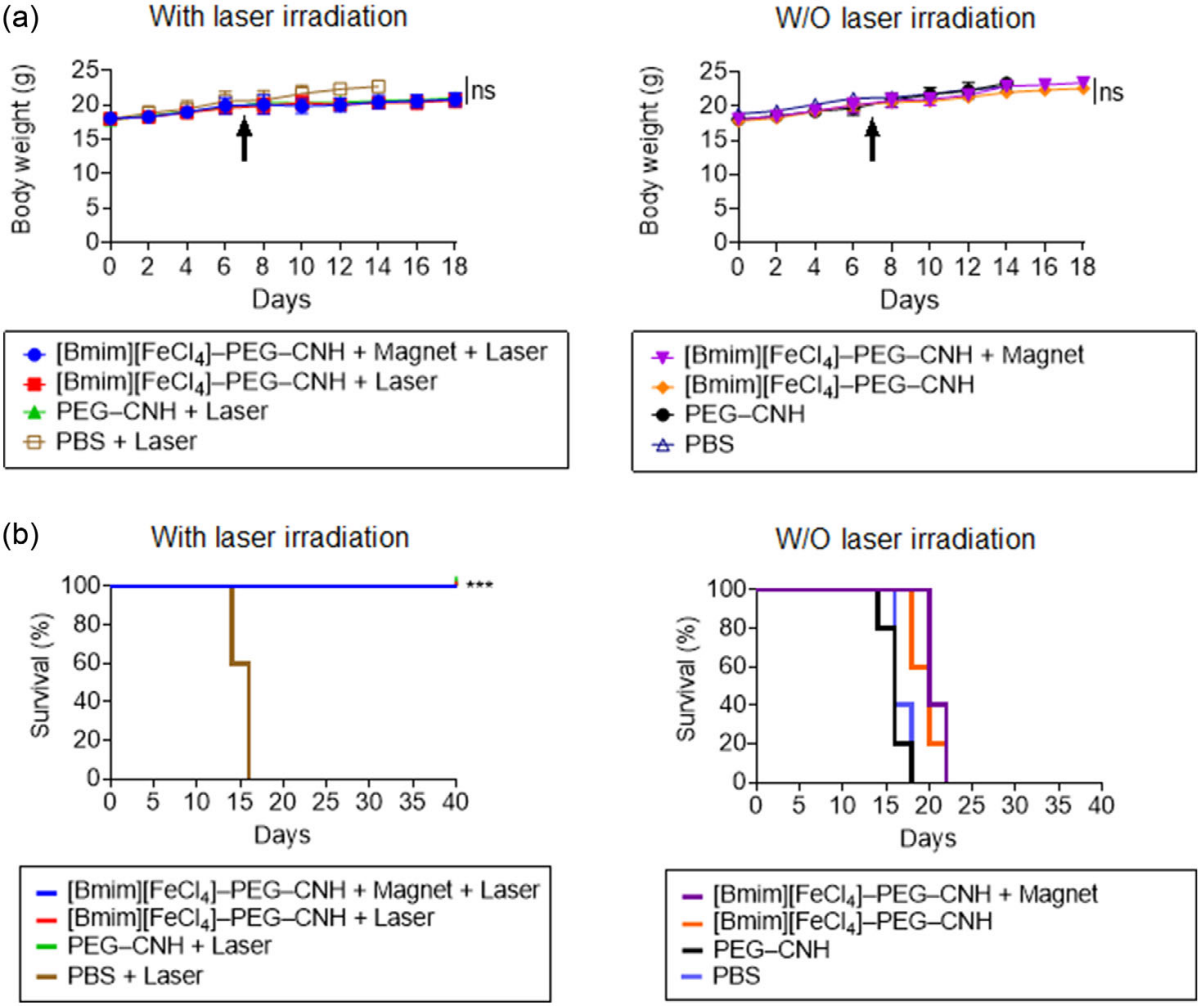
In vivo anticancer efficacy of the nanocomplexes. a) Average body weight of mice during the treatment period. The black arrow indicates the time of sample administration. ns, not significant according to the Student's *t*‐test. b) Kaplan–Meier survival curves of Colon26‐tumor‐bearing mice (*n* = 5 biologically independent mice) after tumor implantation for 30 days. Statistical significance is calculated by comparison with the PBS group. ***p < 0.001 by Log‐rank (Mantel–Cox) test. The [Bmim][FeCl_4_]–PEG–CNH + Magnet + Laser group had a 100% survival rate for at least 40 days.

In addition, the body weights and survival rates of the mice were monitored every second day. In all groups, the body weight of the mice increased steadily, indicating no adverse side effects of the treatment (**Figure** **8**a). Because of the anticancer effects of light‐induced [Bmim][FeCl_4_]–PEG–CNH, the survival rate of mice injected with the nanocomplexes significantly increased, demonstrating the therapeutic effectiveness and safety of [Bmim][FeCl_4_]–PEG–CNH (Figure 8b).

**Figure 9:**
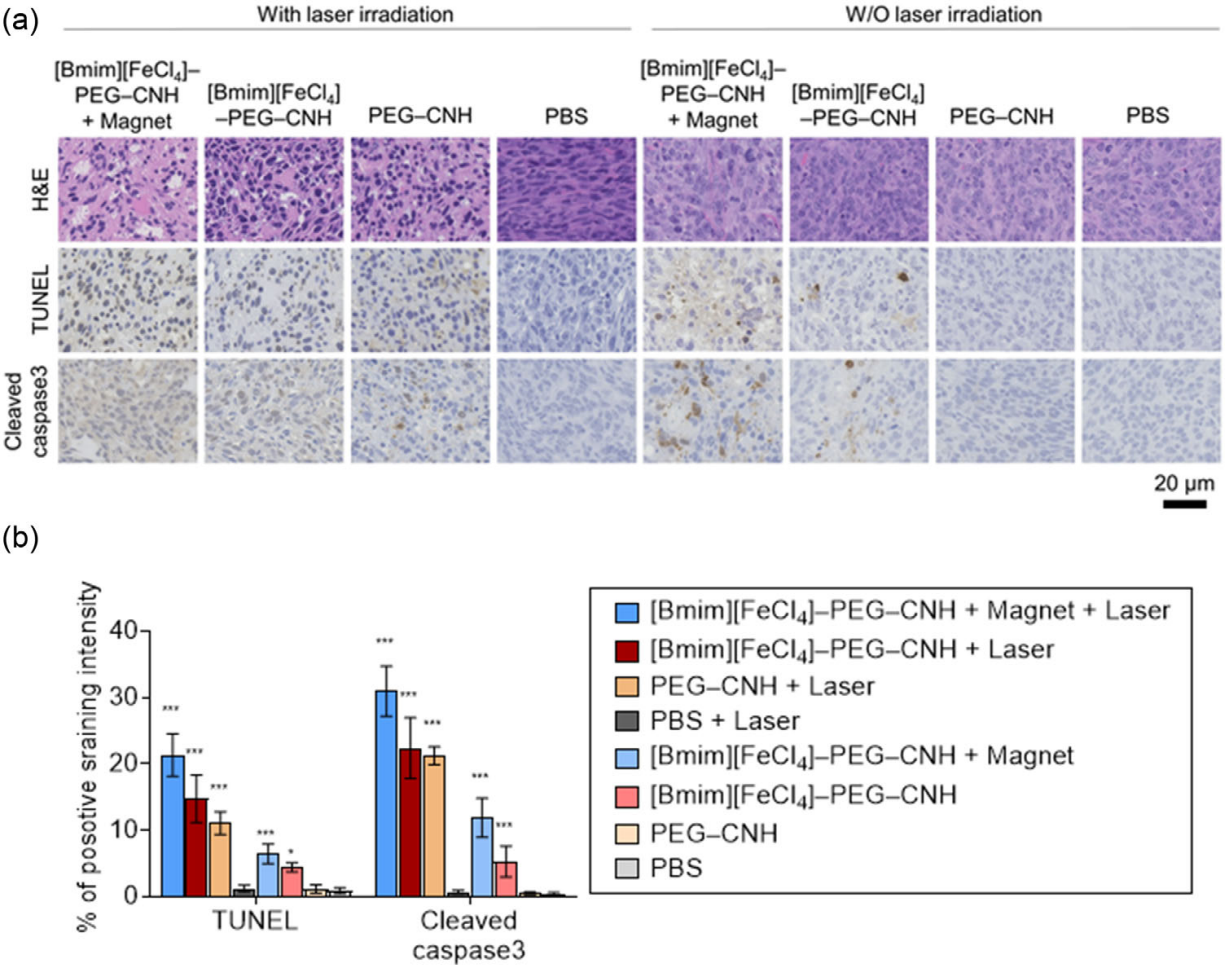
Mechanism of tumor suppression by laser‐ and magnetic field‐induced [Bmim][FeCl_4_]–PEG–CNH nanocomplexes. a) Hematoxylin and eosin (H&E)‐, TUNEL‐, and cleaved caspase‐3 stained tumor tissues collected from various groups of mice on day 1 after their respective treatment. b) Statistical analyses of TUNEL‐ and cleaved caspase‐3‐positively stained tumor tissues are shown in Figure 8a. Data are presented as the mean ± standard error of the mean (SEM); *n* = 7 independent areas (region of interest) in each tumor tissue collected from the mice on day 1 following treatment. Statistical significance is calculated in comparison with the PBS group. *p < 0.05, and ***p < 0.001, by Student's *t*‐test.

The solid tumor suppression behavior of NIR laser‐induced [Bmim][FeCl_4_]–PEG–CNH was further examined using H&E staining, terminal deoxynucleotidyl transferase (TdT)‐mediated 2′‐deoxyuridine, 5′‐triphosphate (dUTP) nick end labeling (TUNEL), and cleaved caspase‐3 staining analysis (**Figure** **9**a). Image‐guided statistical immunohistochemical (IHC) analysis using computer‐aided software was also performed because it is a highly useful and reliable technique for the detection and quantification of target epitopes (e.g., proteins, structures, and cellular components) in a wide variety of tissue types (Figure 9b).^[^ ^57^, ^58^ ^]^ H&E staining revealed that solid tumor cellular structures were destroyed and the cells were fragmented in the [Bmim][FeCl_4_]–PEG–CNH + Magnet, [Bmim][FeCl_4_]–PEG–CNH, and PEG–CNH groups following laser irradiation. The [Bmim][FeCl_4_]–PEG–CNH + Magnet group was the most apparent, indicating a strong antitumor effect. In contrast, no obvious destruction was observed in the PBS‐treated group or any of the non‐irradiated groups. Similarly, based on TUNEL staining results, the [Bmim][FeCl_4_]–PEG–CNH + Magnet with laser irradiation resulted in more apoptotic cells than the other groups. Cleaved caspase‐3 staining confirmed the potent anticancer mechanism of laser‐induced [Bmim][FeCl_4_]–PEG‐CNH in vivo. The [Bmim][FeCl_4_]–PEG–CNH + Magnet and [Bmim][FeCl_4_]–PEG–CNH groups, which were not laser‐irradiated, had few apoptotic cells after TUNEL and cleaved caspase‐3 staining, indicating the anticancer effect of [Bmim][FeCl_4_]. Immunization with lysates from PTT‐treated tumor cells significantly inhibited tumor growth in tumor‐bearing mice.^[^ ^59^, ^60^ ^]^ Herein, the photothermal conversion property of [Bmim][FeCl_4_]–PEG–CNH may effectively induce anticancer immunological responses in the resulting lysates from destroyed cancer cells after laser irradiation, although further experiments are required.

## 3 Conclusion

In this study, we developed chemotherapeutic, optical, and magnetically driven ionic‐liquid‐modified CNH nanocomplexes with excellent water dispersibility and photothermal stability. This study represents the first application of magnetic ionic liquids in cancer treatment. These nanocomplexes exhibited efficient tumor targeting under an external magnetic field and potent photothermal and NIR FL effects for cancer cell theranostics in vitro and in vivo. By improving the precise control of the EPR effect, this approach provides a promising strategy for precise and effective cancer theranostics, potentially advancing the biomedical applications of multifunctional nanomaterials. The in vivo anticancer efficacy of the CNH nanocomplexes was enhanced to suppress tumor growth via a simple and efficient nanoformulation strategy. The efficacy of the proposed CNH nanocomplex system in a syngeneic mouse model was superior to that of previous functional nanoparticles.^[^ ^61^, ^62^, ^63^ ^]^ This simple and effective nanoplatform with synergistic physicochemical pathways has substantial potential for future clinical applications in cancer theranostics, although the proposed nanosystem will require multiple safety tests and the development of an efficient endoscopic laser irradiation system for the treatment of deeper tissues. Moreover, we believe that this nanosystem is more effective than chemotherapeutic, light, and magnetic‐field monotherapies because of the improved rates and durability of responses to combination therapy. We hypothesized that the synergistic strategy of the developed nanosystem would effectively address tumor heterogeneity. Immune modulation induced by this nanosystem presents a new approach for innovative cancer immunotherapy.^[^ ^64^, ^65^ ^]^ The proposed nanocomplex will be useful for designing an effective platform for sophisticated immunological regulation.

Conventional magnetic nanoparticles exhibit high magnetic moments and surface‐area‐to‐volume ratios, which make them suitable for cancer hyperthermia and active‐targeted drug delivery systems.^[^ ^66^ ^]^ Additionally, they can function as contrast agents for magnetic resonance imaging and improve the sensitivity of biosensors and diagnostic tools.^[^ ^66^ ^]^ However, magnetic nanoparticles themselves do not possess anticancer properties unless modified with anticancer drugs. Moreover, the solidity and rigid structures of magnetic nanoparticles are not readily applicable for conjugation with different nanomaterials, such as nanocarbons. In this study, we observed that magnetic ionic liquids exhibit anticancer properties in addition to various unique physicochemical traits for cancer treatment and droplet manipulation. We believe that magnetic ionic liquids potentially offer versatile control owing to their 1) excellent fluidity; 2) adjustable surface tension; 3) chemical controllability and designability; 4) ionic and thermal conductivities; and 5) ability to conjugate with other materials. These comprehensive properties allow magnetic ionic liquids to exhibit controllable molecular functions and make them applicable to various fields, including the electric, magnetic, electromagnetic, wave, light, and biomedical fields. Moreover, the manipulation of magnetic ionic liquids enables intriguing morphological changes and controllable motion (e.g., directional locomotion) of magnetic ionic liquids with new fluidic phenomena and practical applications in soft electronics and robotics in addition to the currently proposed nanomedicine. Exploration of the potential of magnetic ionic‐liquid functions has only recently begun. We hypothesize that the current study will facilitate the further development of innovative applications of magnetic ionic liquids.

## 4 Experimental Section

4.1

4.1.1

##### Nanocomplex Synthesis

The [Bmim][FeCl_4_]–PEG–CNH nanocomplex was prepared as follows. First, 1 mg of CNH (average diameter, ≈80–100 nm; purity, 95%; metal‐free, NEC Corporation, Tokyo, Japan), 1 mg of DSPE–PEG_2000_–NH_2_ (NOF Corporation, Tokyo, Japan), and 200 μL (275.52 mg) of [Bmim][FeCl_4_] (Tokyo Chemical Industry, Tokyo, Japan) were mixed with 10 mL MilliQ water (Direct‐Q UV3, Merck, Darmstadt, Germany) and subjected to pulse‐type sonication (VCX‐600; Sonics, Danbury, CT, USA) for 10 min with crushed ice. The control CNH nanocomplex without magnetism was prepared similarly, except for the addition of [Bmim][FeCl_4_]. The [Bmim][FeCl_4_]–PEG–CNH or PEG–CNH suspension at a high concentration was prepared by increasing the amount of CNH and DSPE–PEG_2000_–NH_2_ at the same ratio.

The ICG‐functionalized [Bmim][FeCl_4_]–PEG–CNH nanocomplex ([Bmim][FeCl_4_]–PEG–ICG–CNH) was prepared as follows. Briefly, 0.1 mg of ICG (Tokyo Chemical Industry) was mixed with 1 mL of the prepared [Bmim][FeCl_4_]–PEG–CNH suspension (CNH concentration =100 μg mL^−1^) and stirred for 1 h under dark conditions. In addition, a highly concentrated [Bmim][FeCl_4_]–PEG–ICG–CNH suspension was prepared by increasing the amounts of [Bmim][FeCl_4_]–PEG–CNH and ICG at the same ratio.

##### Structural and Optical Characterization of the Nanocomplex

The morphology and structure of the [Bmim][FeCl_4_]–PEG–CNH nanocomplex were observed under a high‐resolution transmission electron microscope (TEM) (JEM‐2100Plus, Japan Electron Optics Laboratory, Tokyo, Japan). The hydrodynamic diameter of the [Bmim][FeCl_4_]–PEG–CNH nanocomplex was measured using dynamic light scattering (Zetasier Nano ZS; Malvern Panalytical, UK). The optical absorbance and fluorescence (FL) of the [Bmim][FeCl_4_]–PEG–CNH and [Bmim][FeCl_4_]–PEG–ICG–CNH suspensions were measured by UV‐Vis‐NIR (V‐730 BIO; Jasco, Tokyo, Japan) and FL spectrometry (FP‐8600 NIR Spectrofluorometer; Jasco), respectively. The amounts of PEG and [Bmim][FeCl_4_] conjugated with CNH were estimated by TG analysis (TG‐DTA8122; Rigaku, Tokyo, Japan). TGA traces were recorded for the series using a ramp rate of 5 °C min^−1^ in air. The [Bmim][FeCl_4_]–PEG–CNH and PEG–CNH were synthesized by the same way as the above protocol of nanocomplex synthesis except for using distilled water instead of PBS. The freeze dried [Bmim][FeCl_4_]–PEG–CNH and PEG–CNH was prepared by a freeze dryer (FDU‐1200; EYELA, Tokyo, Japan) before TG measurements.

##### Photothermal Conversion

The [Bmim][FeCl_4_]–PEG–CNH suspension and PBS buffer were irradiated with an 808 nm NIR laser (Civil Laser, Hangzhou, Zhejiang, China) at 0.7 W (≈35.6 mW mm^−2^ and 0.3 W (≈15.3 mW mm^−2^) power (spot diameter ≈ 5 mm) for the indicated conditions. The temperature of the solutions was measured in real‐time using a temperature sensor (AD‐5601A; A&D, Tokyo, Japan). Thermographic images were recorded by infrared thermography (i7, E6; FLIR, Nashua, NH, USA).

The photothermal stability of the [Bmim][FeCl_4_]–PEG–CNH nanocomplex was determined as follows. Briefly, 100 μl of the [Bmim][FeCl_4_]–PEG–CNH suspension was diluted with 1.9 mL of PBS buffer and irradiated with an 808 nm NIR laser for 5 min at 0.7 W (≈35.6 mW mm^−2^, spot diameter ≈3 mm). The optical absorbance spectra were analyzed before and after laser treatment using a UV‐Vis‐NIR spectrometer.

The photothermal conversion efficiency of the nanocomplex was determined according to previously described methods.^[^ ^67^, ^68^ ^]^ The calculation was as follows

(1) $$\eta =hS(T_{\text{Max}}-T_{\text{Surr}})-Q_{\text{Dis}}/I(1-10^{-A_{808}})$$

where *η* is the photothermal conversion efficiency of the nanocomplex, *h* is the heat transfer coefficient, *S* is the surface area of the container, and the value of *hS* is obtained from Equation (2). *T*_Max_ is the maximum steady temperature of the solution of the nanocomplex and *T*_Surr_ is the environmental temperature. *I* and *A*_808_ represent the laser power and the absorbance at 808 nm, respectively. *Q*_Dis_ represents the heat dissipated from the light absorbed by the solvent and the container.

(2) $$hS=m_{D}C_{D}/\tau_{S}$$

where *m*_D_ and *C*_D_ are the mass and the heat capacity of the solvent, respectively. The sample system time constant *τ*_S_ can be calculated by Equation (3).

(3) $$t=-\tau_{S}\ln(\theta )$$

The dimensionless parameter *θ* is introduced as follows

(4) $$\theta =T-T_{\text{Surr}}/T_{\text{Max}}-T_{\text{Surr}}$$

##### Magnetic and Photothermal Locomotion of [Bmim][FeCl_4_]–PEG–CNH Droplet

The [Bmim][FeCl_4_]–PEG–CNH droplet (≈20 μL) was pipetted onto the liquid surface of a dish (diameter 90 mm; ND90‐15, AS ONE, Osaka, Japan) containing 10 mL of 3M Novec 7300 Engineered Fluid (3 M, MN, USA). The magnetic‐driven movement was observed by placing a neodymium magnet obliquely above the droplet (distance ≈ 1.5 cm) and the direction of the movement was adjusted by moving the neodymium magnet. Similarly, the light‐driven movement was observed using an 808 nm NIR laser (0.7 W, ≈35.6 mW mm^−2^; spot diameter ≈ 3 mm) to irradiate the edge of the droplet and the direction of movement was adjusted by changing the direction of laser irradiation.

##### Cell Culture and Viability Test

Murine colon carcinoma (Colon26) cells and normal diploid lineage bud (TIG103) cell lines were obtained from the Japanese Collection of Research Bioresources Cell Bank (Tokyo, Japan). The Colon26 cell line was cultured in Roswell Park Memorial Institute (RPMI) 1640 medium (Nacalai Tesque, Kyoto, Japan) containing 10% fetal bovine serum (FBS), 2 mM of L‐glutamine, 1 mM of sodium pyruvate, gentamycin, and 100 IU mL^−1^ of penicillin‐streptomycin. TIG103 cells were cultured in Eagle's minimal essential medium (Nacalai Tesque) containing 10% FBS, 2 mM of L‐glutamine, 1 mM of sodium pyruvate, gentamycin, 100 IU mL^−1^ penicillin‐streptomycin. The cells were maintained at 37 °C in a humidified chamber containing 5% CO_2_. They were subsequently cryopreserved in liquid nitrogen in multiple vials. The cell stocks were regularly renewed to avoid any genetic instability associated with high passage numbers.

Cell viability was assessed using the Cell Counting Kit‐8 (CCK‐8) assay (Dojindo Laboratories, Kumamoto, Japan) based on the manufacturer's instructions. Briefly, 5000 cells per well were seeded into 96‐well plates, allowed to adhere overnight, exposed to the nanocomplex, and laser‐irradiated as indicated. After washing with fresh medium, the cells were incubated with 10 μL of CCK‐8 for 3 h at 37 °C in a humidified chamber containing 5% CO_2_. The absorbance at 450 nm was determined using a microplate reader (Infinite M200 PRO; Tecan, Männedorf, Switzerland).

##### Intracellular Penetration of the Nanocomplex

Colon26 cells (2.5 × 10^5^ cells per dish) were seeded in poly‐L‐lysine‐coated 35 mm glass‐bottom dishes (Matsunami glass, Osaka, Japan) and allowed to adhere overnight. The cells were exposed to the [Bmim][FeCl_4_]–PEG–CNH (CNH concentration = 0.1 mg mL^−1^) suspension for 24 h at 37 °C in a humidified incubator containing 5% CO_2_. After washing thoroughly with fresh PBS buffer, Colon26 cells were observed using a microscope equipped with a mirror unit (IRDYE800‐33LP‐A‐U01; Semrock, Lake Forest, IL, USA) and an objective lens (×40 magnification, aperture 0.95; UPLSAPO20X, Olympus, Tokyo, Japan) at room temperature. For FL bioimaging, Colon26 cells were incubated with the [Bmim][FeCl_4_]–PEG–ICG–CNH suspension (CNH concentration = 0.1 mg mL^−1^, ICG concentration = 0.1 mg mL^−1^) in a similar manner. After washing with PBS buffer, the cells were examined and images were acquired using an FL microscope (BZ‐X800, Keyence, Tokyo, Japan).

##### Direct Observation of Laser‐Induced Cancer Cell Destruction

Colon26 cells (2.5 × 10^5^ cells per dish) were seeded in 35 mm glass‐bottom dishes and cultured overnight. The [Bmim][FeCl_4_]–PEG–CNH suspension (CNH concentration = 100 μg mL^−1^) or fresh PBS buffer was added to the dishes for 24 h. After washing three times with PBS buffer, the cells were maintained in RPMI medium. The destruction of the cancer cells triggered by the laser‐induced nanocomplex using the laser irradiation setup was performed as follows: An 808 nm, 254 mW (≈129 mW mm^−2^) NIR laser beam from a continuous‐wave diode laser (Sigma Koki, Tokyo, Japan) was incorporated into a microscope (IX73, Olympus). The laser beam (spot diameter ≈ 50 μm) was focused onto the target position (×40 magnification; aperture 0.95; UPLSAPO40X, Olympus) at room temperature for 3 s. The videos were recorded using an electron‐multiplying, charge‐coupled device camera system (DP80, Olympus) before and during irradiation.

##### In vivo Anticancer Therapy

The animal experiments were conducted following the protocols approved by the Institutional Animal Care and Use Committee of the Japan Advanced Institute of Science and Technology (JAIST) (No. 04‐007). All the mice were obtained from Japan SLC (Hamamatsu, Japan) (female; 5 weeks old; *n* = 40; average weight = 16 g; BALB/cCrSlc). Colon26 cell‐derived mouse tumors were generated by injecting 100 μL of Matrigel Matrix (mixed with RPMI medium, v/v = 1:1; Dow Corning, Corning, NY, USA) containing 1 × 10^6^ cells into the dorsal right side of the mice. After ≈1 week, when the tumor volume reached ≈100 mm^3^, the mice were intravenously injected with 200 μL of [Bmim][FeCl_4_]–PEG–CNH suspension (CNH concentration = 1 mg mL^−1^, [Bmim][FeCl_4_] concentration = 10 μL mL^−1^), PEG–CNH suspension (CNH concentration = 1 mg mL^−1^), or fresh PBS buffer. In particular, for the magnetic groups, the neodymium magnet (diameter ≈ 6 mm; magnetic flux density ≈ 230 mT, Okazaki, Wakayama, Japan) was fixed on the tumor top with a medical bandage (NICHIBAN, Tokyo, Japan). The dorsal right‐side tumors were irradiated for 5 min every day 24 h after sample injection (a total of four laser irradiation sessions) using an 808 nm laser (0.7 W, ≈35.6 mW mm^−2^, spot diameter ≈ 5 mm). Thermographic measurements were carried out during irradiation using infrared thermography. Tumor formation and overall health (vitality and body weight) were monitored every other day. Tumor volume was calculated using the formula *V* = *L* × *W* ^2^/2, where *L* and *W* denote the length and width of the tumor, respectively. When the tumor volumes reached 1500 mm^3^, the mice were euthanized according to JAIST Institutional Animal Care and Use Committee guidelines.

##### In vivo Fluorescence Bioimaging

To monitor the chronological changes in FL intensity resulting from the [Bmim][FeCl_4_]–PEG–ICG–CNH nanocomplex tumor‐targeting effect, Colon26 tumor‐bearing mice (female; 6 weeks old; *n* = 4; average weight = 18 g; average tumor size = 100 mm^3^; BALB/cCrSlc) were injected intravenously with 200 μL of PBS or [Bmim][FeCl_4_]–PEG–ICG–CNH nanocomplex suspension (ICG concentration = 1 mg mL^−1^, CNH concentration = 1 mg mL^−1^). The mice were euthanized and the major organs, including the heart, liver, spleen, lungs, and kidneys, as well as the tumor, were imaged using an in vivo FL imaging system (VISQUE InVivo Smart‐LF, Vieworks, Anyang, Republic of Korea) with a 3 s exposure time and an ICG filter (Ex, 740–790 nm; Em, 810–860 nm) at 4, 12, and 24 h postinjection. The FL images were acquired and analyzed using CleVue software.

##### Immunohistochemistry Staining of Tumor Tissues

Colon26 tumor‐bearing mice (*n* = 4) were euthanized on the day after intravenous injection and laser irradiation. Subsequently, tumor tissue from the treatment groups was collected for immunohistochemistry (IHC) staining. IHC was performed by the Biopathology Institute Co., Ltd. (Oita, Japan) using standard protocols. Briefly, the primary tumors were surgically removed, fixed in 10% formalin, processed for paraffin embedding, and cut into 3–4 μm sections. After incubation with primary antibody (Table S3, Supporting Information), the sections were stained with hematoxylin and eosin and examined by light microscopy (IX73, Olympus). The areas showing positive staining in the tumor tissues were analyzed using a light microscopy system (BZ‐X800, Keyence) and hybrid cell count and microcell count software (Keyence).

##### Safety Tests

The complete blood count (CBC) was measured using a Celltac α blood cell counting machine (Microsemi LC‐712; HORIBA, Japan), and biochemical parameters were investigated by Oriental Yeast Co. (Tokyo, Japan). BALB/cCrSlc mice (female; 6 weeks; *n* = 5; average weight = 18 g; Japan SLC, Inc.) were injected in the tail vein with PBS buffer (200 μL) or [Bmim][FeCl_4_]–PEG–CNH nanocomplex (200 μL, CNH concentration = 1 mg mL^−1^. Blood samples were collected from the inferior vena cava of each mouse after 7 days.

Vital organ tissues from the different treatment groups were harvested after nanocomplex administration for 7 days for histological staining. All surgeries were performed under anesthesia and all efforts were made to relieve suffering. The histology of liver, spleen, heart, lungs, and kidney was analyzed using a light microscopy system (BZ‐X800). H&E staining was performed by the Biopathology Institute Co., Ltd. Briefly, excised organ tissues were fixed in 10% neutral buffered formalin for at least 48 h, then dehydrated with increasing concentrations of ethanol and embedded in paraffin. Tissue sections of 3–4 μm thick were attached to positively charged glass slides and stained with H&E.

##### Statistical Analysis

All experiments were performed in triplicate and repeated three or more times. Quantitative values are expressed as the mean ± standard error of the mean (SEM) of at least three independent experiments. Statistical differences were identified by a Student's *t*‐test or Log‐rank (Mantel‐Cox) test using GraphPad Prism software, version 9.4.0 (GraphPad Software, Boston, MA, USA). A *p* value < 0.05 was considered statistically significant.

## Conflict of Interest

The authors declare no conflict of interest.

## Author Contributions

**Yun Qi**: data curation (lead); formal analysis (lead); investigation (lead); validation (lead); writing—original draft (lead). **Eijiro Miyako**: data curation (supporting); formal analysis (supporting); funding acquisition (lead); investigation (supporting); methodology (lead); project administration (lead); resources (lead); software (lead); supervision (lead); validation (supporting); visualization (lead); writing—original draft (lead); writing—review & editing (lead).

## Supporting information

Supplementary Material

## Data Availability

The data that support the findings of this study are available from the corresponding author upon reasonable request.
