# Supplementary material for: Multifunctional Magnetic Ionic Liquid‐Carbon Nanohorn Complexes for Targeted Cancer Theranostics
Source: Small Sci. 2025 Mar 3;5(5):2400640. doi: 10.1002/smsc.202400640 (PMC12087772; doi:10.1002/smsc.202400640)
Supplement: Supplementary file 1 — Supplementary Material [file SMSC-5-2400640-s001.zip › smsc12702-sup-0001-SuppData-S1.pdf]

Supporting Information

Chemotherapeutic, Light- and Magnetic Field-Controllable Multifunctional Nanocomplexes for Targeted Cancer Theranostics

*Yun Qi and Eijiro Miyako**

Graduate School of Advanced Science and Technology, Japan Advanced Institute of Science and Technology, 1-1 Asahidai, Nomi, Ishikawa 923-1292, Japan

*Corresponding author: E-mail: e-miyako@jaist.ac.jp

Supporting Video Legends

Supporting Video S1. Magnetic locomotion of the [Bmim][$FeCl_4$]–PEG–CNH droplet.

Supporting Video S2. Photothermal locomotion of the [Bmim][$FeCl_4$]–PEG–CNH droplet.

Supporting Video S3. Laser-induced [Bmim][$FeCl_4$] droplet.

Supporting Video S4. Destruction of cancer cells by laser-induced [Bmim][$FeCl_4$]–PEG–CNH.

Supporting Video S5. Control cancer cells without [Bmim][$FeCl_4$]–PEG–CNH after laser irradiation.

Supporting Figures and Tables

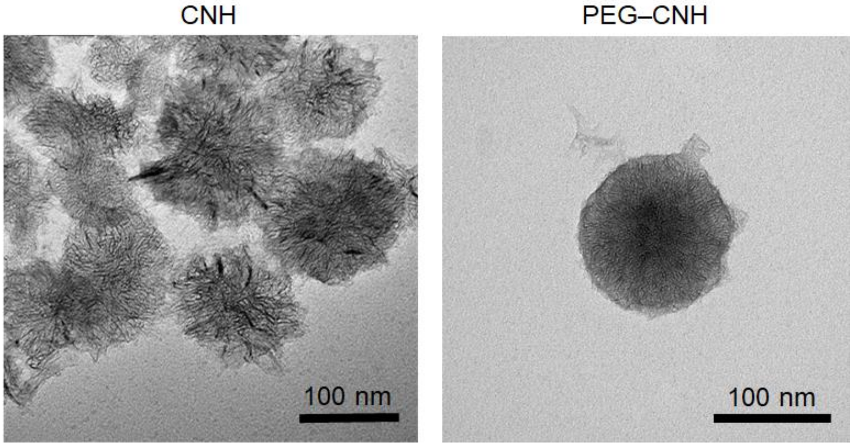

Figure S1. TEM images of CNH (left) and PEG–CNH (right).

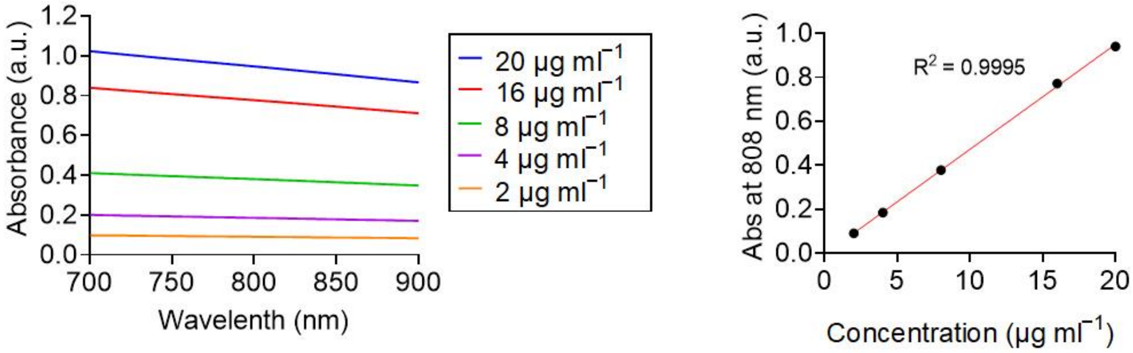

Figure S2. Absorbance at 808 nm versus concentration for the [Bmim][$FeCl_4$]–PEG–CNH.

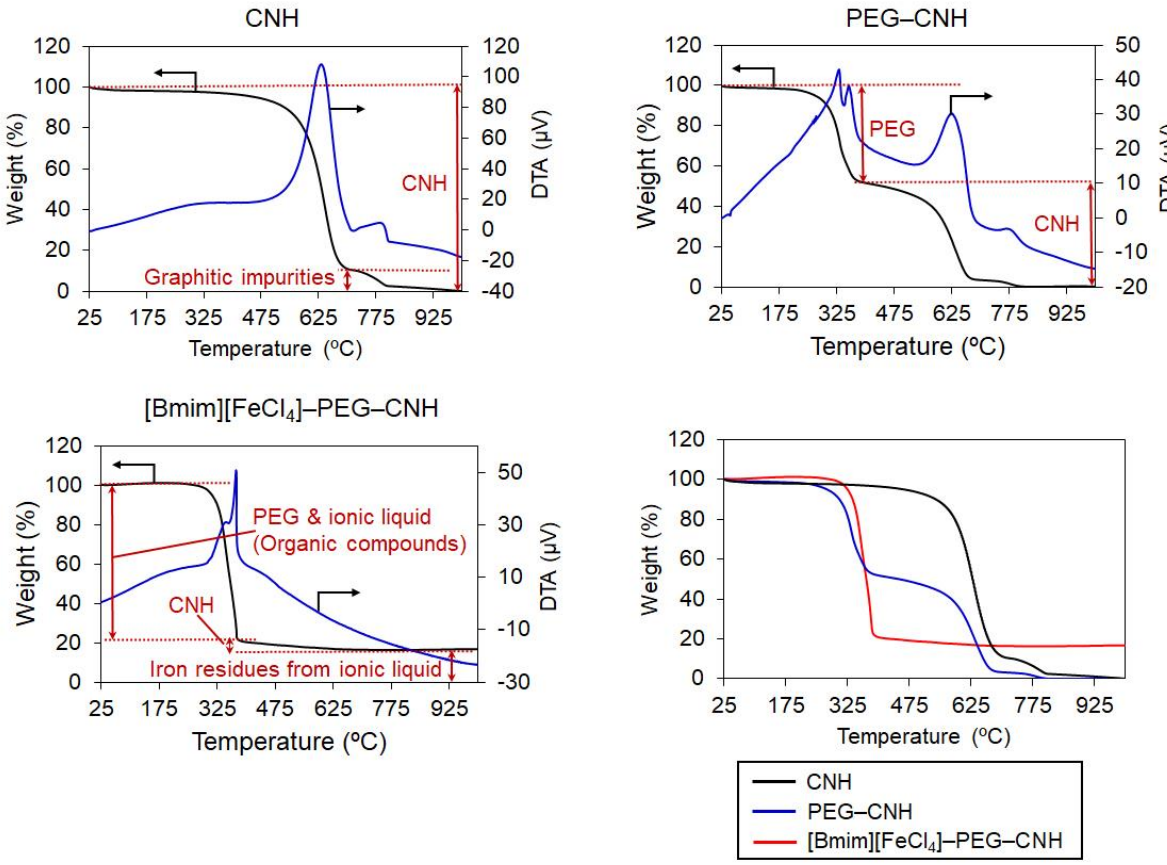

Figure S3. Thermogravimetric analysis (TGA) of CNH, PEG–CNH, and [Bmim][$FeCl_4$]–PEG–CNH.

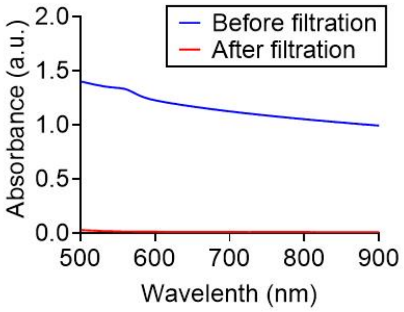

Figure S4. Ultraviolet–visible–near-infrared (UV–vis–NIR) absorption spectram of the filtrate of [Bmim][$FeCl_4$]–PEG–CNH. The [Bmim][$FeCl_4$]–PEG–CNH aqueous suspension was filterd (0.22 µm-polytetrafluoroethylene syringe filter, Osaka Chemical, Osaka, Japan) before the measurement.

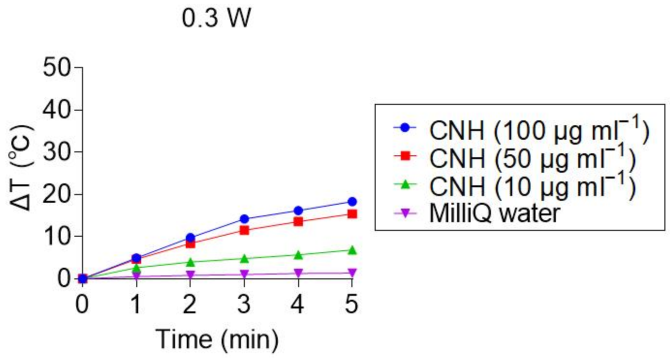

Figure S5. Laser-induced temperature increase in MilliQ water (control) and [Bmim][$FeCl_4$]–PEG–CNH suspension at different CNH concentrations by 808 nm laser irradiation at 0.3 (~15.3 mW mm^{-2}). Data are presented as means ± standard error of the mean (SEM) (n = 3; independent tests).

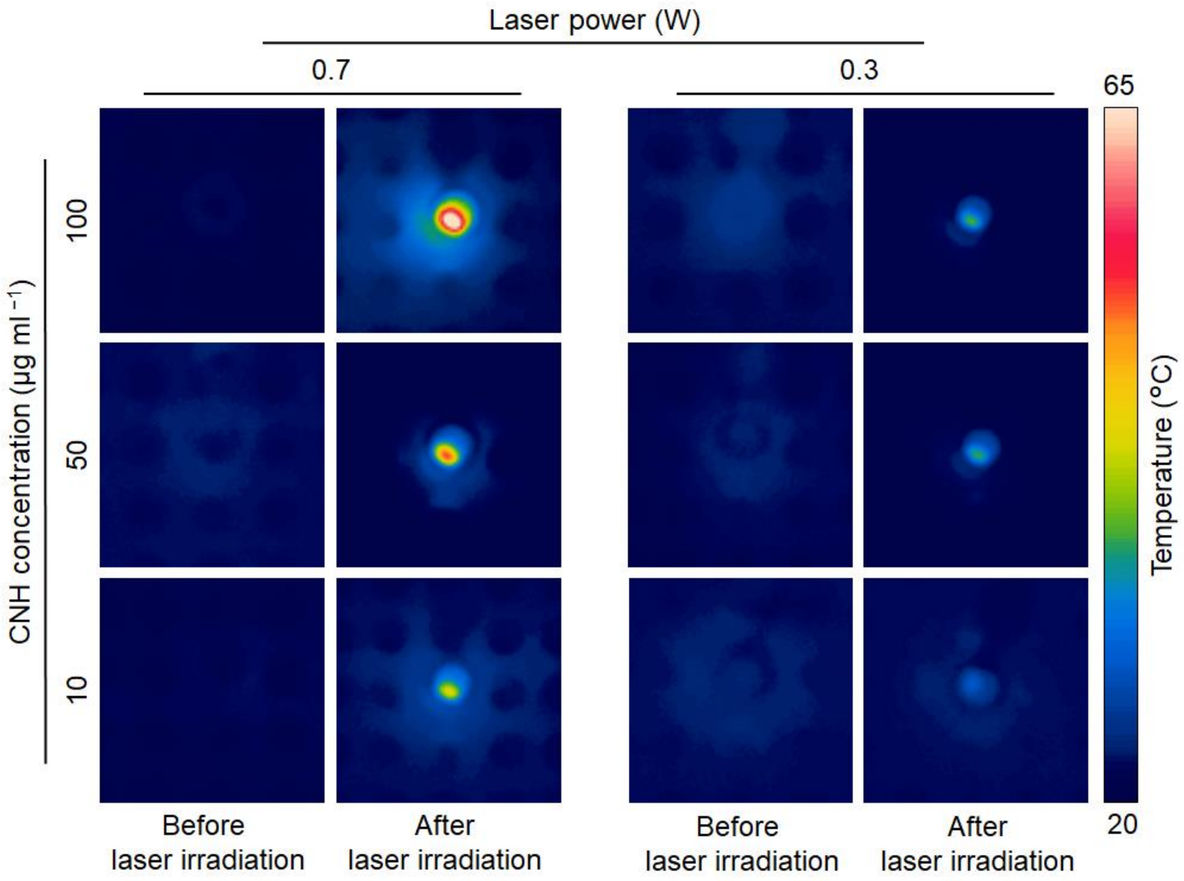

Figure S6. Thermographic images of various concentrations of [Bmim][$FeCl_4$]–PEG–CNH suspension after 5 min of laser irradiation at NIR laser powers of 0.7 W (~35.6 mW mm^{-2}) and 0.3 W (~15.3 mW mm^{-2}).

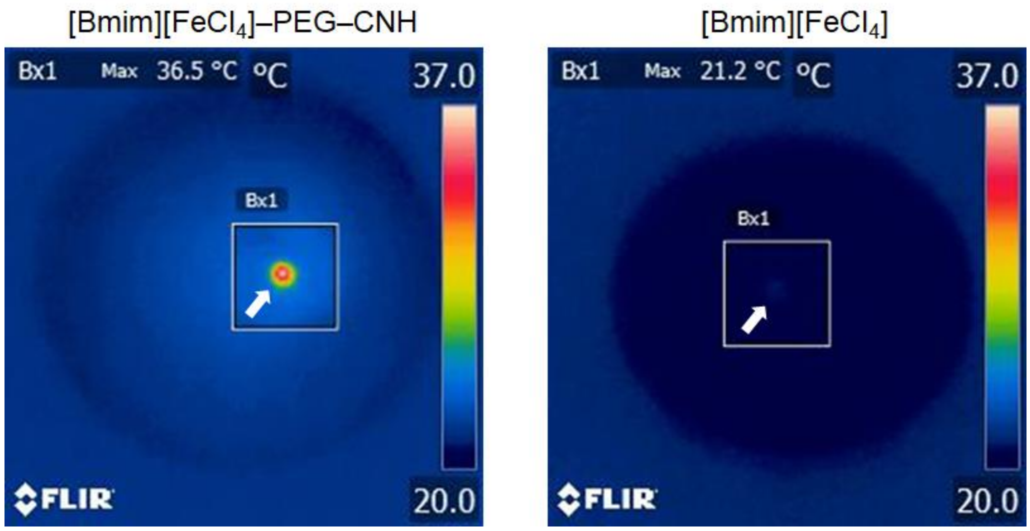

Figure S7. Thermographic image of [Bmim][$FeCl_4$]–PEG–CNH and [Bmim][$FeCl_4$] droplet in a fluorous solvent after laser irradiation at NIR laser powers of 0.7 W (~35.6 mW mm^{-2}). The white arrows represent the location of [Bmim][$FeCl_4$]–PEG–CNH and [Bmim][$FeCl_4$] droplets.

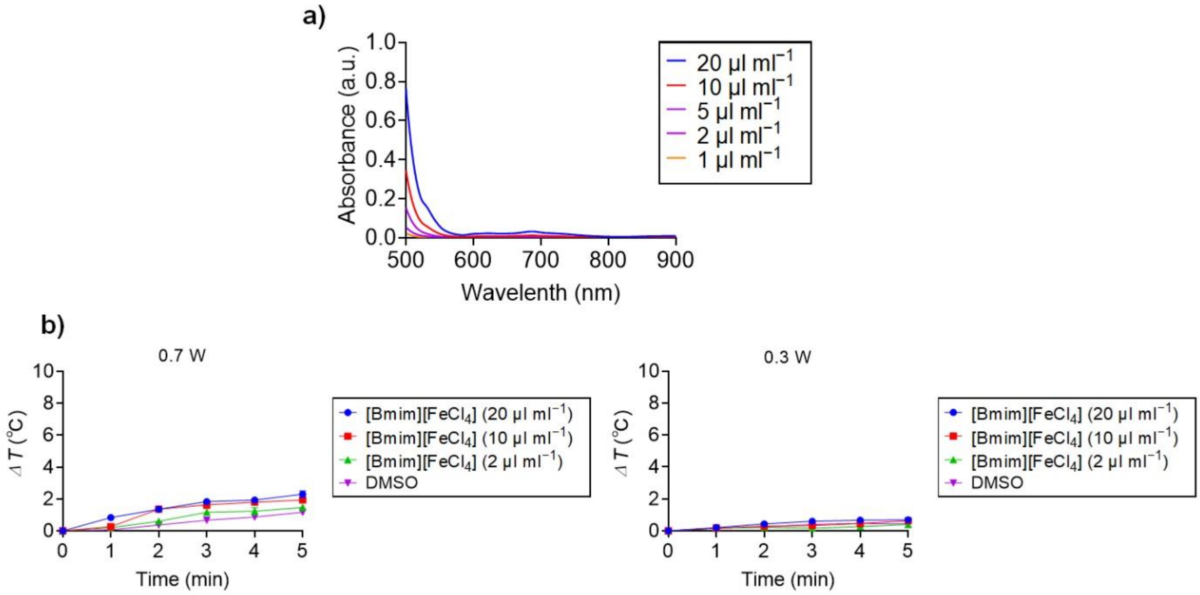

Figure S8. Photothermal conversion property of [Bmim][$FeCl_4$]. a) UV–vis–NIR absorbance spectra of DMSO solution of [Bmim][$FeCl_4$] at different [Bmim][$FeCl_4$] concentration. b) Laser-induced temperature increase in [Bmim][$FeCl_4$] suspension at different [Bmim][$FeCl_4$] concentrations by 808 nm laser irradiation at 0.7 W (~35.6 mW mm^{-2}) or 0.3 W (~15.3 mW mm^{-2}). Data are presented as means ± standard error of the mean (SEM) (n = 3; independent tests).

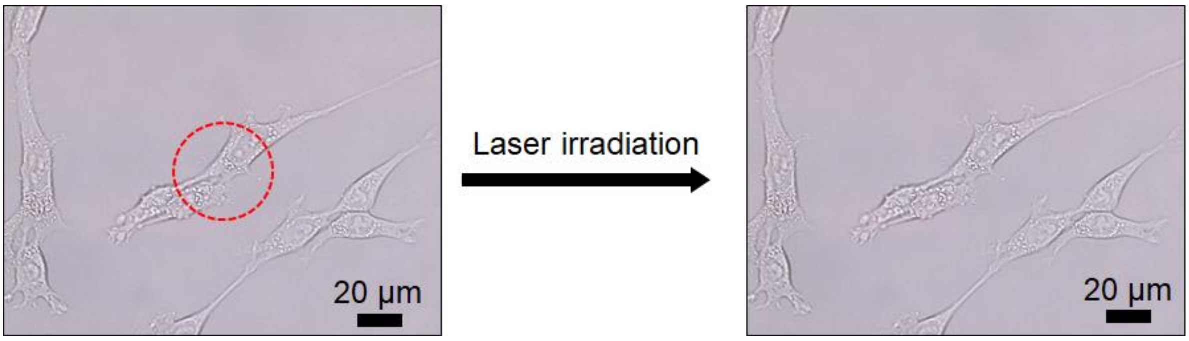

Figure S9. Colon26 cancer cell behavior in PBS before and after laser irradiation [Wavelength = 808 nm, Laser power = 254 mW (~129 mW mm^{-2})]. The red dashed circle represents the location of the laser irradiation.

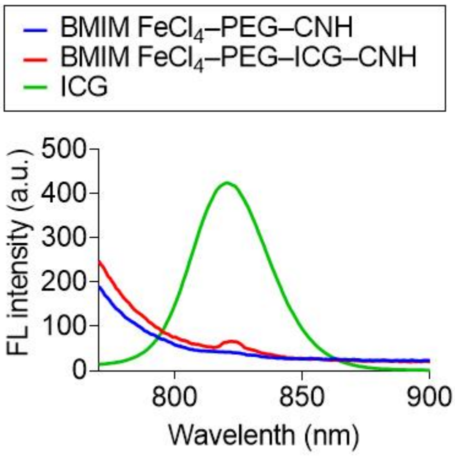

Figure S10. FL spectra of the [Bmim][FeCl4]–PEG–ICG–CNH, [Bmim][FeCl4]–PEG–CNH, and ICG suspension at a 750 nm excitation wavelength. (ICG concentration = 125 μg ml^{-1}, CNH concentration = 125 μg ml^{-1}).

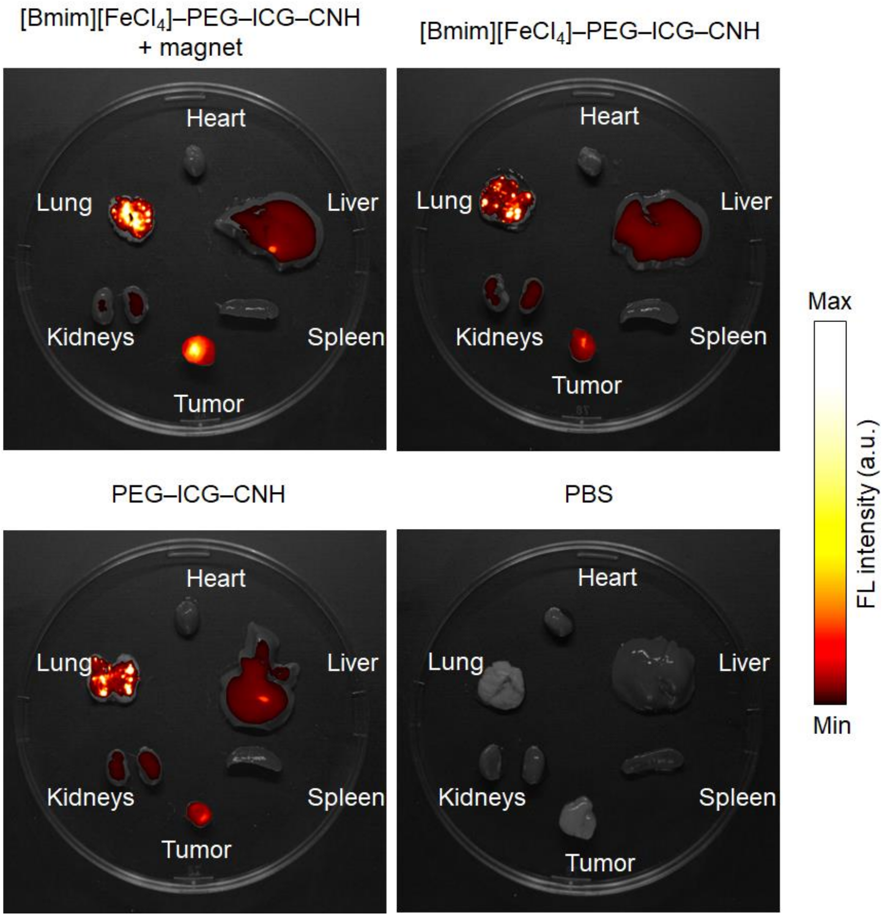

Figure S11. FL imaging of the extracted vital organs of Colon26 tumor-bearing mice after intravenous injection of [Bmim][$FeCl_4$]–PEG–ICG–CNH, PEG–ICG–CNH, and PBS buffer. (ICG concentration = 1 mg ml^{-1}, CNH concentration = 1 mg ml^{-1}).

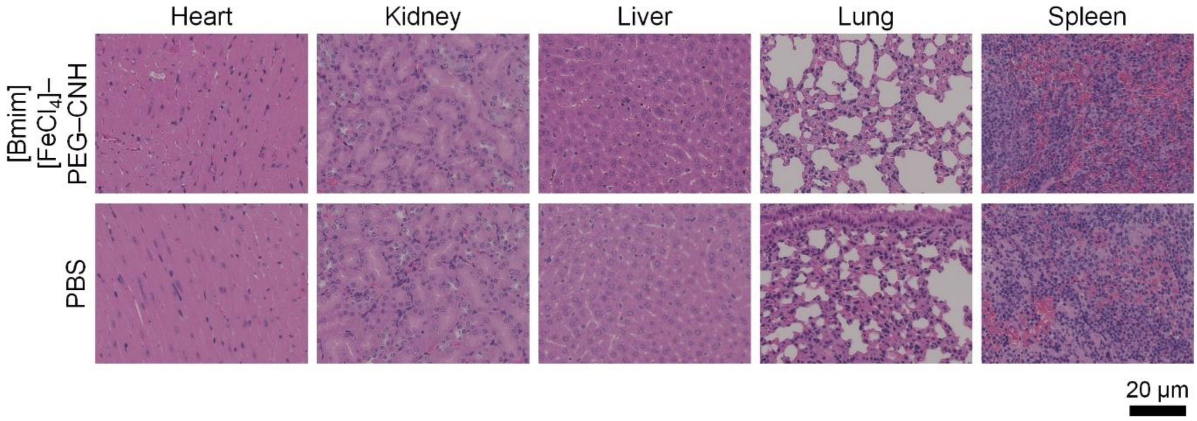

Figure S12. H&E staining in conventional organs sectioned after i.v. injection of [Bmim][$FeCl_4$]–PEG–ICG–CNH or PBS after 7 days.

Table S1. The photothermal conversion efficiency of materials in previous reports

Material	Photothermal conversion efficiency (%)	Reference
[Bmim][$FeCl_4$]–PEG–ICG–CNH	63	This study
Gold nanorods	21	42
Gold nanoshells	13	42
Copper selenide	22	43
Carbon dots	31	44
Semiconducting polymer nanoparticles	37	9

Table S2. CBCs and biochemical parameters of the mice injected with PBS or [Bmim][$FeCl_4$]–PEG–ICG–CNH nanoparticle dispersion after 7 days.

Measured value	Entry	Unit	PBS (n = 6)	[Bmim][$FeCl_4$]–PEG–ICG–CNH (n = 6)	P value
CBC	WBC	$\times 10^2$ /μL	65.8 ± 3.85	61.8 ± 7.73	> 0.05
	RBC	$\times 10^4$ /μL	869.2 ± 28.25	874.7 ± 22.88	> 0.05
	HGB	g/dL	15.0 ± 0.43	15.3 ± 0.28	> 0.05
	HCT	%	15.5 ± 1.39	15.6 ± 0.75	> 0.05
	MCV	fL	52.3 ± 0.68	52.1 ± 0.62	> 0.05
	MCH	pg	17.3 ± 0.29	17.5 ± 0.30	> 0.05
	MCHC	g/dL	33.0 ± 0.30	33.5 ± 0.21	> 0.05
	PLT	$\times 10^4$ /μL	83.3 ± 3.43	86.4 ± 8.69	> 0.05
Biochemical parameters	TP	g/dL	4.6 ± 0.15	4.7 ± 0.15	> 0.05
	ALB	g/dL	3.0 ± 0.12	3.1 ± 0.08	> 0.05
	BUN	mg/dL	26.1 ± 2.43	23.5 ± 1.68	> 0.05
	CRE	mg/dL	0.1 ± 0.01	0.1 ± 0.01	> 0.05
	Na	mEq/L	144.3 ± 1.25	143.8 ± 0.69	> 0.05
	K	mEq/L	22.3 ± 1.32	23.0 ± 0.70	> 0.05
	Cl	mEq/L	104.5 ± 1.80	103.3 ± 1.80	> 0.05
	AST	IU/L	46.7 ± 5.47	44.7 ± 1.11	> 0.05
	ALT	IU/L	21.0 ± 3.46	20.5 ± 0.76	> 0.05
	LDH	IU/L	246.0 ± 59.66	239.2 ± 23.69	> 0.05
	AMY	IU/L	2378.3 ± 192.85	2662.7 ± 107.20	> 0.05
	CK	IU/L	65.5 ± 10.31	53.0 ± 4.51	> 0.05

Data are represented as means ± standard errors of the mean (SEM.); n = 6 biologically independent mice. Statistical analyses comprise the Student's two-sided *t test*.

Abbreviations: ALB, albumin; ALT, alanine transaminase; AMY, amylase; AST, aspartate aminotransferase; BUN, blood urea nitrogen; Cl, chlorine; CK, creatine kinase; CRE, creatinine; HCT, hematocrit; HGB, hemoglobin; K, potassium; LDH, lactate dehydrogenase; MCH, mean corpuscular hemoglobin; MCHC, mean corpuscular hemoglobin concentration; MCV, mean corpuscular volume; Na, sodium; PLT, platelet; RBC, red blood cell; TP, total protein; WBC, white blood cell.

Table S3. Antibodies used in this study.

Antibody	Type	Source	Catalog No.	Application
Anti-digoxigenin-peroxidase	Sheep Polyclonal	Merck Millipore	S7100	Tunel
Caspase-3	Rabbit Polyclonal	Cell Signaling Technology	9661S	IHC (1:100)
